# Supplementary material for: MtrA modulates Mycobacterium tuberculosis cell division in host microenvironments to mediate intrinsic resistance and drug tolerance
Source: Cell Rep. 2023 Aug 4;42(8):112875. doi: 10.1016/j.celrep.2023.112875 (PMC10480492; doi:10.1016/j.celrep.2023.112875)
Supplement: Document S1. Figures S1–S6 and Tables S1 and S2 [file mmc1.pdf]

Cell Reports, Volume *42*

Supplemental information

MtrA modulates *Mycobacterium tuberculosis* cell division in host microenvironments to mediate intrinsic resistance and drug tolerance

Eliza J.R. Peterson, Aaron N. Brooks, David J. Reiss, Amardeep Kaur, Julie Do, Min Pan, Wei-Ju Wu, Robert Morrison, Vivek Srinivas, Warren Carter, Mario L. Arrieta-Ortiz, Rene A. Ruiz, Apoorva Bhatt, and Nitin S. Baliga

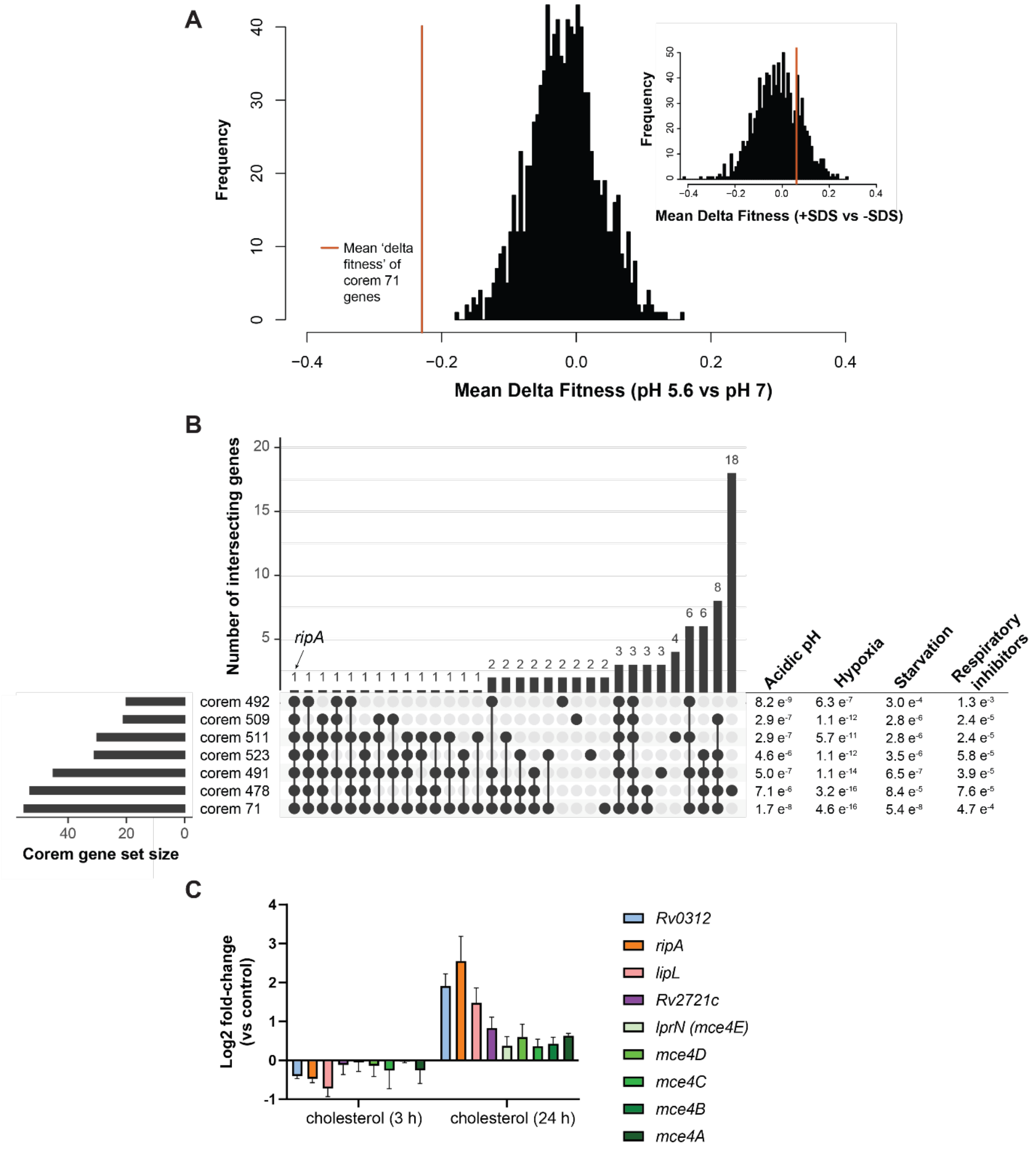

Figure S1. Environmental influences of corem 71. Related to Figure 2. (A) Histogram of mean delta fitness between acidic pH and neutral pH from 1000 permutations to generate shuffled gene sets. In each permutation, the produced shuffled gene set had the same size as corem 71. The orange line represents the observed value for corem 71. Inset displays the results of same analysis with mean delta fitness between the presence and absence of 0.05% SDS. (**B**) Upset plot of genes

in corems with similar environmental influences. Graph plots the number of genes in each corem and the intersection of genes within multiple corems. Each column corresponds to a single corem (single dot) or a set of corems (dots connected by lines below the X axis) containing the same genes. The number of genes in each set appears above the column, while the corems shared are indicated in the graphic below the column, with the corem numbers on the left. The enrichment (*P*-value) of environmental influences for each corem, calculated by hypergeometric test with BH correction is shown on the right. (**C**) Quantification (mean ± s.d., $n = 3$ biological replicates) of corem 532 genes' mRNA levels by microarray. H37Rv cells were grown in the presence of cholesterol for indicated time and compared to control. Data is from Yang Liu and Gary Schoolnik's unpublished carbon sources data.

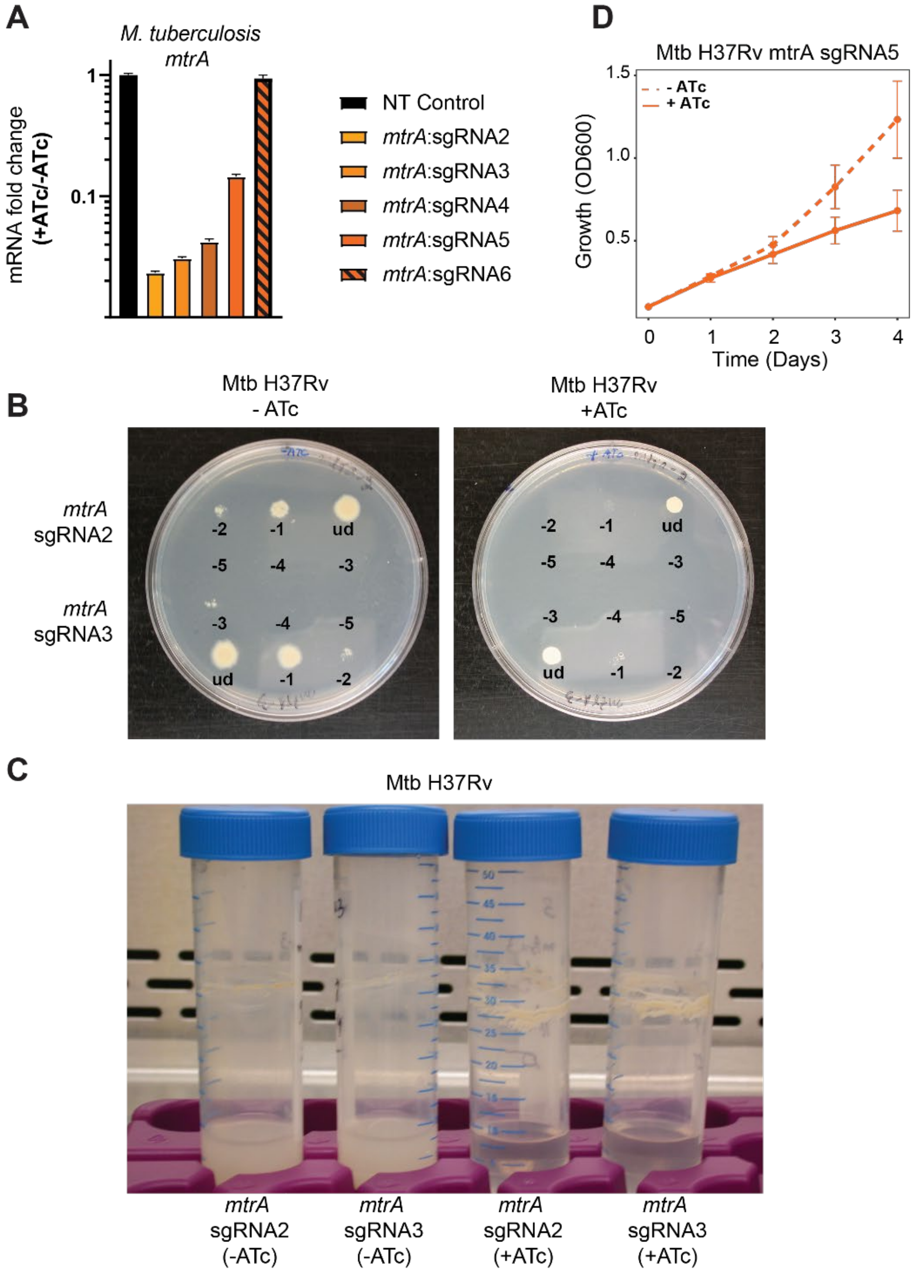

Figure S2. CRISPRi knockdown of *mtrA* with PAMs of various "strengths" in *M. tuberculosis*. Related to Figure 2. (**A**) Quantification (mean ± SD, *n* = 3 biological replicates) of *mtrA* mRNA levels by RT-qPCR. Strains were grown ± ATc for 4 days before collecting RNA. (**B**) Serial 10-fold dilutions of *M. tuberculosis* H37Rv CRISPRi strains with sgRNA2 and sgRNA3 targeting *mtrA* were spotted on 7H10 agar plates with (+ATc) or without ATc (-ATc control). (**C**)

Photographs of liquid cultures of the indicated strains. (**D**) Growth of *M. tuberculosis* H37Rv CRISPRi strain with sgRNA5 in liquid 7H9-rich media with (solid line) or without (dotted line) ATc. Growth was monitored daily by optical density at 650 nm. Points are the average of three biological replicates and error bars represent standard deviation.

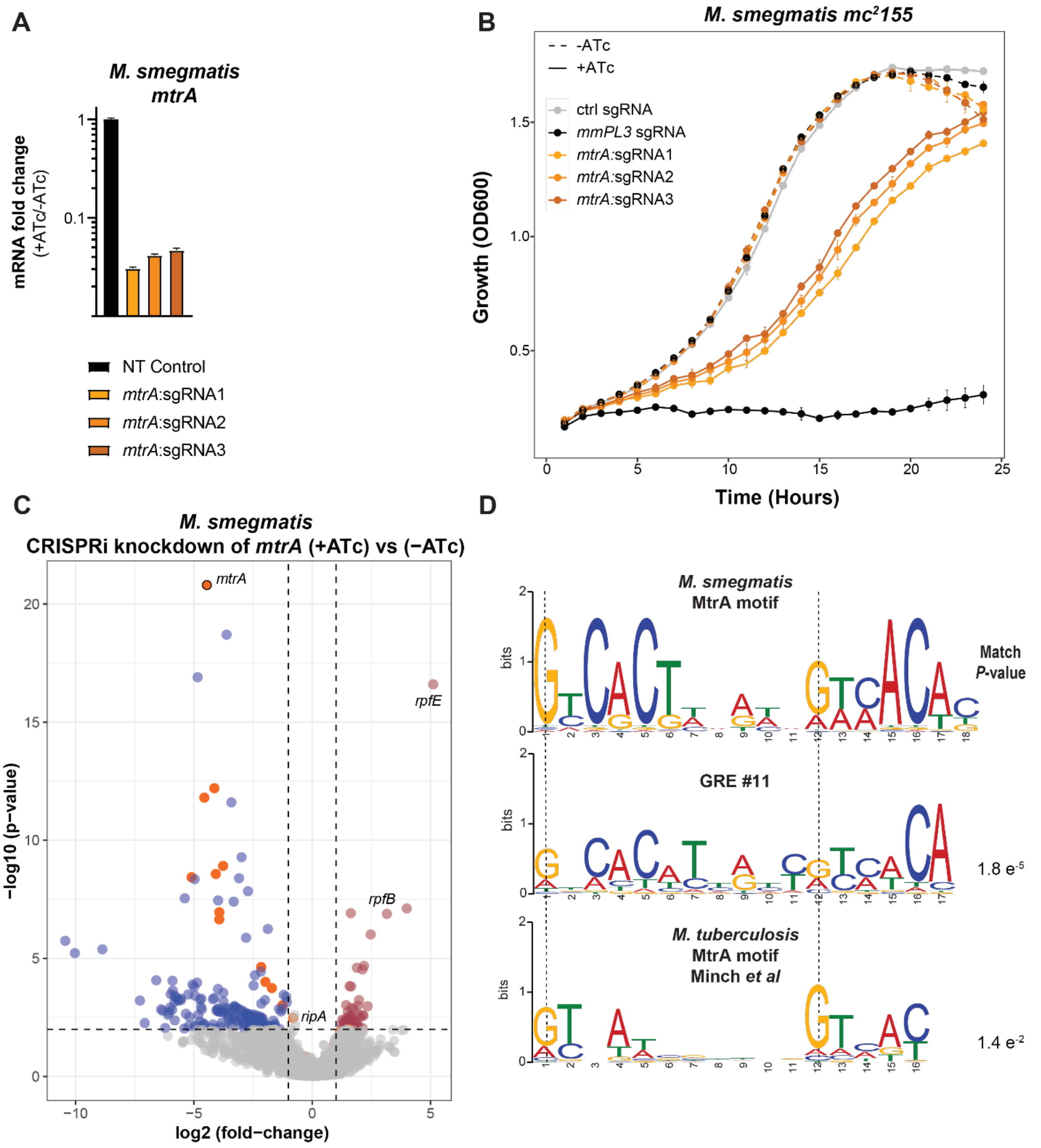

Figure S3. CRISPRi knockdown of *mtrA* in *M. smegmatis.* Related to Figure 2. (**A**) Quantification (mean ± SD, *n* = 3 biological replicates) of *mtrA* mRNA levels by RT-qPCR. Strains were grown ± ATc overnight before collecting RNA. (**B**) Growth of *M. smegmatis* mc^{2}155 CRISPRi strains with sgRNA1, sgRNA2, sgRNA3, control (ctrl) sgRNA, and sgRNA targeting

the essential gene *mmPL3* in liquid 7H9-rich media with (solid line) or without (dotted line) ATc. Growth was monitored hourly by optical density at 650 nm. Points are the average of three biological replicates and error bars represent standard deviation. (**C**) Volcano plot of differentially expressed genes for induced vs uninduced CRISPRi knockdown of *mtrA* in *M. smegmatis*. The significantly differentially expressed genes were selected by *p*-value < 0.01 and absolute log2 fold-change > 1. Dots represent different genes, with labels for particular genes of interest. Grey dots are genes without significant different expression, red dots are significantly up-regulated genes (*N* = 58 genes) and blue dots are significantly down-regulated genes (*N* = 185 genes). The orange dots are all genes of corem 71. (**D**) MEME analysis was performed on the promoter regions of candidate genes found to be significantly downregulated upon *mtrA* knockdown in Msm (*n* = 17 genes). The motif logo of MtrA from Msm compared to GRE #11 and MtrA motif deciphered through analysis of ChIP-seq mapped binding locations in Mtb [S1]. The *P*-values from alignment carried out with Tomtom [S2] are shown.

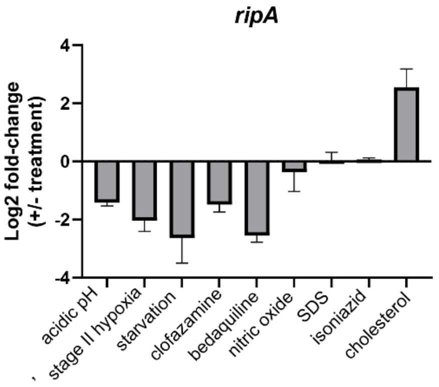

Condition	Description	Reference
acidic pH	pH 5.6 vs pH 7.0	This study
stage II hypoxia	0% dissolved oxygen, after 2 day slow/controlled oxygen depletion	PMID: 32348771
starvation	2 weeks in PBS-tyloxapol vs log growth	Baliga lab unpublished data
clofazamine	0.6 µg/mL clofazimine treated for 24 h vs untreated	GEO: GSE165673
bedaquiline	3.5 µg/mL bedaquiline treated for 24 h vs untreated	GEO: GSE165673
nitric oxide	1 mM DETA/nitric oxide for 24 h vs log growth	PMID: 28811595
SDS	0.05% SDS treated for 24 h vs untreated	Baliga lab unpublished data
Isoniazid	1.8 µg/mL isoniazid treated for 24 h vs untreated	GEO: GSE165673
cholesterol	1 mg/mL cholesterol for 24 vs control	Yang Liu and Gary Schoolnik's unpublished carbon sources data

Figure S4. Conditional expression of MtrA regulatory target, *ripA*. Related to Figure 2. The expression of *ripA* in conditions significantly enriched in corem 71 —acidic pH, hypoxia, starvation and treatment with respiratory inhibitors (*e.g.*, clofazimine and bedaquiline). Other conditions not enriched in corem 71 (*e.g.*, nitric oxide, SDS, and isoniazid treatment) are included for comparison. RipA is also found in corem 532 that is enriched in growth on cholesterol. Details of experimental conditions and data source are given in the table to the right.

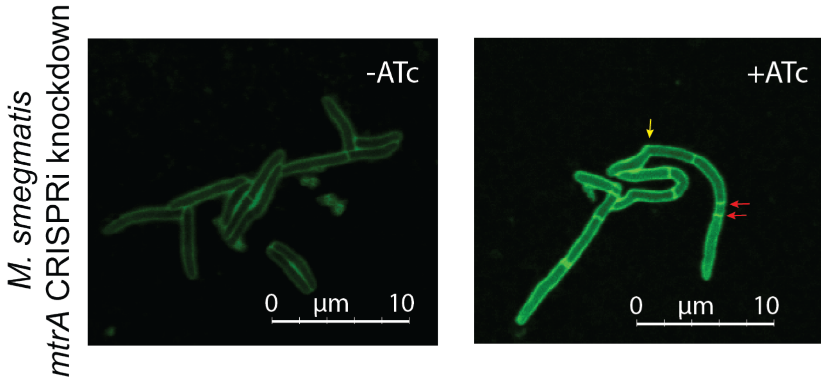

Figure S5. MtrA controls cell division in *M. smegmatis*. Related to Figure 4. Example micrographs of uninduced (-ATc) and induced (+ATc) CRISPRi knockdown of *mtrA* with sgRNA1 in Msm. After knockdown, cells were labeled with HCC-amino-D-alanine (HADA) for 3.5 h. Red arrows indicate multiple septa and yellow arrow indicates the curved shape phenotype. Data are representative of at least two independent experiments.

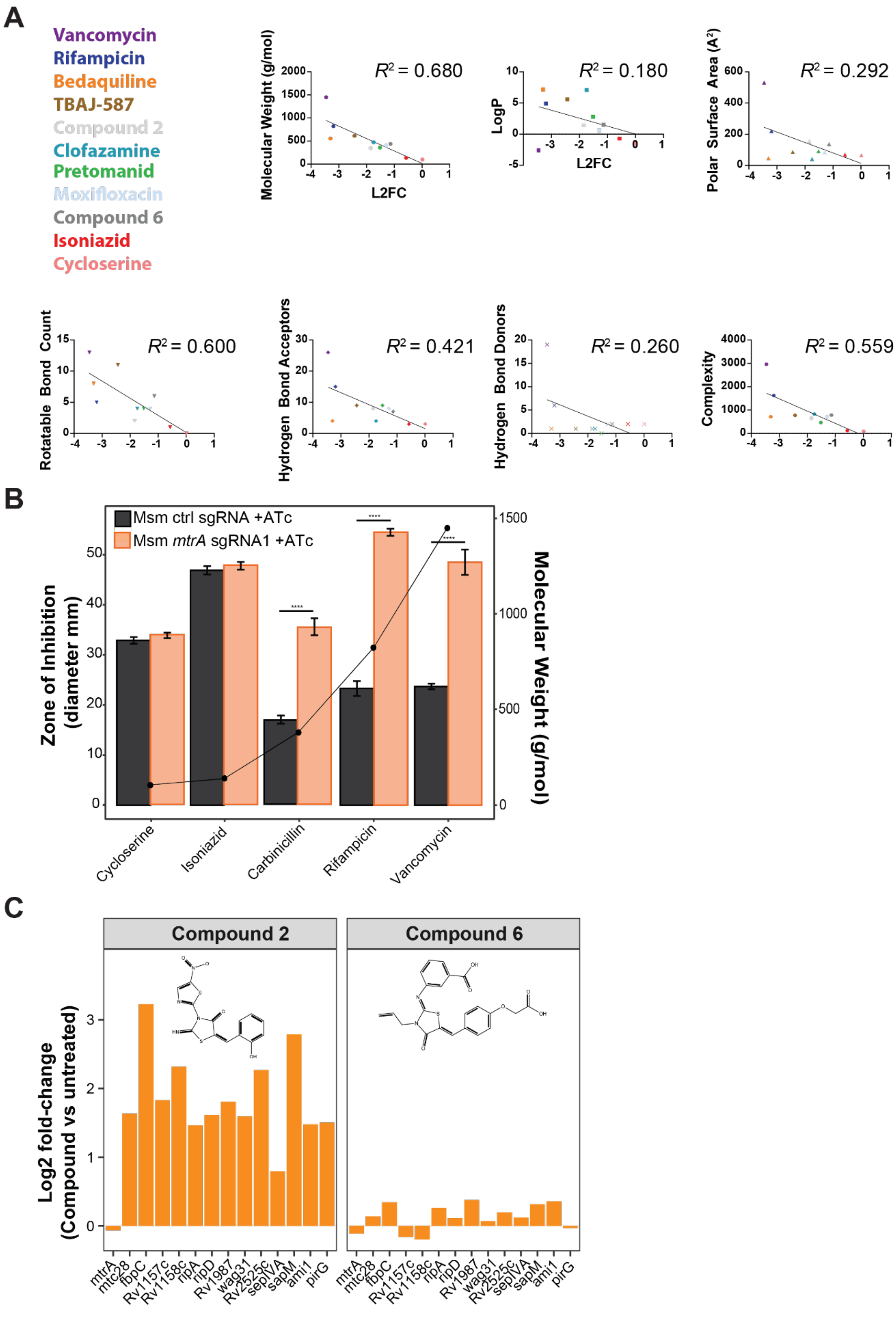

A
Vancomycin
Rifampicin
Bedaquiline
TBAJ-587
Compound 2
Clofazamine
Pretomanid
Moxifloxacin
Compound 6
Isoniazid
Cycloserine
Molecular Weight (g/mol)
R^2 = 0.680
L2FC
LogP
R^2 = 0.180
Polar Surface Area (Å²)
R^2 = 0.292
Rotatable Bond Count
R^2 = 0.600
Hydrogen Bond Acceptors
R^2 = 0.421
Hydrogen Bond Donors
R^2 = 0.260
Complexity
R^2 = 0.559
B
Msm ctrl sgRNA +ATc
Msm *mtrA* sgRNA1 +ATc
Zone of Inhibition (diameter mm)
Molecular Weight (g/mol)
Cycloserine
Isoniazid
Carbinicillin
Rifampicin
Vancomycin
C
Compound 2
Compound 6
Log2 fold-change (Compound vs untreated)
mtrA
mtc28
fbpC
Rv1157c
Rv1158c
ripA
ripD
Rv1987
wag31
Rv2525c
sepIVA
sapM
ami1
pirG

Figure S6. Antibiotic activity with *mtrA* knockdown. Related to Figure 5. (**A**) For each drug, the Log2 fold-change (L2FC) in IC_{50} for *mtrA* knockdown in Mtb compared to control (NT sgRNA) was compared to various physiochemical properties. The IC_{50} measurements were calculated using a nonlinear fit and the R^2 values were determined using linear regression in GraphPad Prism. (**B**) The induced CRISPRi *mtrA* knockdown with sgRNA1 and control (ctrl) sgRNA strains of Msm were spread on LB plates containing 100 ng/ml ATc inducer in triplicate. A filter disc with 10 µl antibiotic was place in the center of plate and the diameter of inhibition of growth was measured after 4 days of growth. Antibiotic concentration on disc: cycloserine (100 mg/ml); isoniazid (0.5 mg/ml); carbinicillin (100 mg/ml); rifampicin (0.5 mg/ml); vancomycin (6 mg/ml). Significance was determined by Student's T-test. ****: p-value < 0.0001. Results are mean from technical triplicates. All data are representative of at least two independent experiments. (**C**) Mtb H37Rv was treated with 34 µg/mL of Compound 2 or 80 µg/mL of Compound 6 for 72 h. Cell pellets were collected for RNA extraction and mRNA was quantitated by RNA-seq.

Table S1. **sgRNAs used in this study**.

Organism	Gene	sgRNA name	Forward	Reverse	PAM (5'-3')
M. smegmatis	*mtrA*	sgRNA1	GGGAGGTGTCGAAACCCTCACCAC	AAACGTGGTGAGGGTTTCGACACC	AGAAC
M. smegmatis	*mtrA*	sgRNA2	GGGAATCGATGCCGTTCATCCCAG	AAACCTGGGATGAACGGCATCGAT	AGCAT
M. smegmatis	*mtrA*	sgRNA3	GGGAGACGGTGTCGGTCTTGGCGG	AAACCCGCCAAGACCGACACCGTC	AGCAT
M. smegmatis	NT	control_sgRNA	GGGAGGAGACGATTAATGCGTCTCG	AAACCGAGACGCATTAATCGTCTCC	AGAAA
M. smegmatis	*mmpL3*	mmpL3	GGGAGCGACAGACTGGCTGCCCTCGTC	AAACGACGAGGGCAGCCAGTCTGTCGC	AGAAA
M. tuberculosis	*mtrA*	sgRNA2	GGGAATCCACGGTGTCGGTCTTTGCGG	AAACCCGCAAAGACCGACACCGTGGAT	AGCAT
M. tuberculosis	*mtrA*	sgRNA3	GGGAGATTTCTACGTCGGCGATGG	AAACCCATCGCCGACGTAGAAATC	AGCAT
M. tuberculosis	*mtrA*	sgRNA4	GGGAGTCTTTGCGGTGAGCATCACGA	AAACTCGTGATGCTCACCGCAAAGAC	GGAAC
M. tuberculosis	*mtrA*	sgRNA5	GGGAGCCGGTAACCCCATACCTGTT	AAACAACAGGTATGGGGTTACCGGC	AGCAG
M. tuberculosis	*mtrA*	sgRNA6	GGGAGTCAGCACCACAGTCGGGTTC	AAACGAACCCGACTGTGGTGCTGAC	GGGAT
M. tuberculosis	NT	control_sgRNA	GGGAGGAGACGATTAATGCGTCTCG	AAACCGAGACGCATTAATCGTCTCC	AGAAA

Table S2. Genes with predicted regulation by MtrA.

Gene	Name	Description	Gene with reduced fitness at acidic pH	Gene with GRE #11 in promoter	Mtb *mtrA* knockdown +Atc vs –ATc Log2 FC (p <0.01)	Mtb *mtrA* knockdown Li *et al* Log2 FC (p <0.01)	Msm homolog	Msm *mtrA* knockdown +Atc vs –ATc Log2 FC (p <0.05)
Rv3246c	*mtrA*	essential response regulator			-3.76	-1.21	*MSMEG_1874*	-4.56
Rv0040c	*mtc28*	secreted protein	X	X	-2.99	-3.58	***MSMEG_6919***	**-4.13**
Rv0129c	*ag85C/fbpC*	cell wall trehalose dimycolate biosynthesis	X	X	-1.59	-2.6	***MSMEG_3580***	**-3.77**
Rv0179c	*lprO*	lipoprotein		X	-1.08	-1.5	***MSMEG_0210***	**-1.7**
Rv0312		proline and threonine rich		X	-2.68	-4.66	*MSMEG_0638*	ns
Rv1157c		conserved protein		X	-1.24	-0.85	***MSMEG_5153***	**-4.45**
Rv1158c		conserved protein	X	X	-1.9	-2.71	***MSMEG_5151***	**-5.1**
Rv1435c		proline, valine, glycine rich secreted		X	-2.85	-2.24	*MSMEG_0673*	-3.93
Rv1477	*ripA*	peptidoglycan endopeptidase	X	X	-2.4	-1.81	***MSMEG_3145***	**-0.80**
Rv1566c	*ripD*	peptidoglycan endopeptidase		X	-2.54	-2.17	*MSMEG_3477*	-3.1
Rv1690	*lprJ*	lipoprotein		X	-1.72	-2.55	***MSMEG_4689***	**-4.07**
Rv1987		probable chitinase		X	-3.3	-2.21		
Rv2145c	*wag31*	essential cell elongation protein DivIVA		X	-1.2	-0.66	*MSMEG_4217*	ns
Rv2525c		peptidoglycan glycoside hydrolase		X	-1.07	-0.69	*MSMEG_6815*	ns
Rv2894c	*xerC*	tyrosine recombinase		X	-2.26	-1.68	***MSMEG_2515***	**-1.26**
Rv2927c	*sepIVA*	essential cell septation protein		X	-1.2	ns	***MSMEG_2416***	**1.11**
Rv3310	*sapM*	acid phosphatase	X	X	ns	-1.24		
Rv3717	*ami1*	peptidoglycan amidase	X	X	-1.75	-1.46	***MSMEG_6281***	**-3.92**
Rv3810	*pirG/erp*	exported repetitive protein	X	X	-3.0	-1.67	***MSMEG_6405***	**-1.98**
Rv1075c		conserved exported protein			-1.82	-1.53	*MSMEG_5272*	ns
Rv1076	*lipU*	lipase			-4.91	-3.11	*MSMEG_5271*	ns
Rv1469	*ctpD*	cation transporter P-type ATPase		*	-2.00	-1.51	*MSMEG_5403*	-1.55
Rv1478	*ripB*	peptidoglycan endopeptidase	X		-3.13	-2.37	*MSMEG_3146*	-1.20
Rv1754c		conserved protein			-2.92	-1.78	*MSMEG_2107*	-3.62
Rv3229c	*desA3*	stearoyl-CoA 9-desaturase			-1.03	-1.34	*MSMEG_1886*	-0.93

Msm homologs in bold have significant MtrA binding site in their promoter. Genes shaded in grey are not part of corem 71.

Supplemental Information References

S1 Minch, K.J., Rustad, T.R., Peterson, E.J., Winkler, J., Reiss, D.J., Ma, S., Hickey, M., Brabant, W., Morrison, B., Turkarslan, S., et al. (2015). The DNA-binding network of Mycobacterium tuberculosis. Nat Commun *6*, 5829. 10.1038/ncomms6829.

S2 Gupta, S., Stamatoyannopoulos, J.A., Bailey, T.L., and Noble, W.S. (2007). Quantifying similarity between motifs. Genome Biol *8*, R24. 10.1186/gb-2007-8-2-r24.
