# Supplementary material for: MtrA modulates Mycobacterium tuberculosis cell division in host microenvironments to mediate intrinsic resistance and drug tolerance
Source: Cell Rep. 2023 Aug 4;42(8):112875. doi: 10.1016/j.celrep.2023.112875 (PMC10480492; doi:10.1016/j.celrep.2023.112875)
Supplement: Document S2. Article plus supplemental information [file mmc6.pdf]

Cell Reports

Article

MtrA modulates *Mycobacterium tuberculosis* cell division in host microenvironments to mediate intrinsic resistance and drug tolerance

Graphical abstract

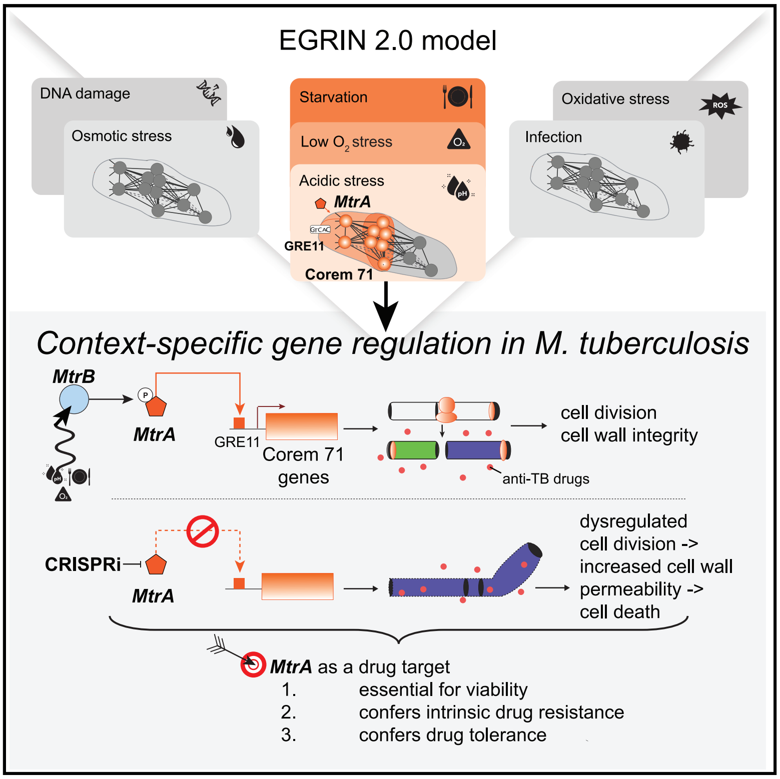

Highlights

- EGRIN 2.0 models environmental context-specific gene regulation in *M. tuberculosis*
- EGRIN 2.0 delineates how MtrAB tunes Mtb cell division in specific environments
- MtrAB regulation confers intrinsic resistance to and tolerance of anti-TB drugs
- MtrA is also essential for Mtb, making it an important target for TB drug development

Authors

Eliza J.R. Peterson, Aaron N. Brooks, David J. Reiss, ..., Rene A. Ruiz, Apoorva Bhatt, Nitin S. Baliga

Correspondence

eliza.peterson@isbscience.org (E.J.R.P.),
nitin.baliga@isbscience.org (N.S.B.)

In brief

The success of *Mycobacterium tuberculosis* (Mtb) emerges from its capability to adapt to complex and varying microenvironments within the host. Peterson et al. develop an EGRIN 2.0 model to facilitate exploration of conditional gene regulation and drug targets that operate in specific host microenvironments.

Peterson et al., 2023, Cell Reports *42*, 112875
August 29, 2023 © 2023 The Author(s).
https://doi.org/10.1016/j.celrep.2023.112875

CellPress

Cell Reports

CellPress
OPEN ACCESS

Article

MtrA modulates *Mycobacterium tuberculosis* cell division in host microenvironments to mediate intrinsic resistance and drug tolerance

Eliza J.R. Peterson,[1,*] Aaron N. Brooks,[1,7] David J. Reiss,[1] Amardeep Kaur,[1] Julie Do,[1] Min Pan,[1] Wei-Ju Wu,[1] Robert Morrison,[2] Vivek Srinivas,[1] Warren Carter,[1] Mario L. Arrieta-Ortiz,[1] Rene A. Ruiz,[1] Apoorva Bhatt,[3] and Nitin S. Baliga[1,4,5,6,8,*]
[1]Institute for Systems Biology, Seattle, WA 98109, USA
[2]Laboratory of Malaria, Immunology and Vaccinology, National Institute of Allergy and Infectious Diseases, NIH, Bethesda, MD 20892, USA
[3]School of Biosciences and Institute of Microbiology and Infection, University of Birmingham, Birmingham B15 2TT, UK
[4]Departments of Biology and Microbiology, University of Washington, Seattle, WA 98195, USA
[5]Molecular and Cellular Biology Program, University of Washington, Seattle, WA 98195, USA
[6]Lawrence Berkeley National Lab, Berkeley, CA 94720, USA
[7]Present address: Ginkgo Bioworks, Boston, MA 02210, USA
[8]Lead contact
*Correspondence: eliza.peterson@isbscience.org (E.J.R.P.), nitin.baliga@isbscience.org (N.S.B.)
https://doi.org/10.1016/j.celrep.2023.112875

SUMMARY

The success of *Mycobacterium tuberculosis* (Mtb) is largely attributed to its ability to physiologically adapt and withstand diverse localized stresses within host microenvironments. Here, we present a data-driven model (EGRIN 2.0) that captures the dynamic interplay of environmental cues and genome-encoded regulatory programs in Mtb. Analysis of EGRIN 2.0 shows how modulation of the MtrAB two-component signaling system tunes Mtb growth in response to related host microenvironmental cues. Disruption of MtrAB by tunable CRISPR interference confirms that the signaling system regulates multiple peptidoglycan hydrolases, among other targets, that are important for cell division. Further, MtrA decreases the effectiveness of antibiotics by mechanisms of both intrinsic resistance and drug tolerance. Together, the model-enabled dissection of complex MtrA regulation highlights its importance as a drug target and illustrates how EGRIN 2.0 facilitates discovery and mechanistic characterization of Mtb adaptation to specific host microenvironments within the host.

INTRODUCTION

Upon infection with *Mycobacterium tuberculosis* (Mtb), interactions between the pathogen and the immune system create microenvironments that are compositionally distinct and changing over time.[1] As Mtb navigates these microenvironments, the bacterium relies on gene-regulatory networks (GRNs) to swiftly transition physiological states to adapt from one niche to another. An accurate and comprehensive GRN model of Mtb would inform on the transcriptional programs employed by the pathogen in distinct microenvironments. Furthermore, a fully characterized GRN model would enable actionable hypotheses for disrupting networks leading to physiological adaptations that interfere with treatment efficacy.[2] Thus, GRN models can shed light on the heterogeneity of Mtb physiology created by local microenvironments and potentially identify opportunities for new tuberculosis (TB) treatment.

The GRN structure is encoded in an organism's genome as transcription factor (TF) binding sites, referred to as gene regulatory elements (GREs). GREs, ~6–20 nt DNA sequences, are invariant to environmental stresses. However, environmental cues (and genetic perturbations) alter the affinity of TFs to bind GREs, which in turn modulates transcription in condition-specific manners. Therefore, the goal of reconstructing a GRN is to produce an unbiased genome-wide map of GREs, including information about what regulators bind to those sequences, in what environments they are bound, the consequences of TF-GRE binding on activating or repressing transcription of downstream genes, and, importantly, how TF binding throughout the genome ultimately influences cellular physiology.

We previously reported models of *Escherichia coli* and *Halobacterium salinarum*[3] that realized these goals of GRN inference. The models were constructed with the EGRIN 2.0 inference methodology, which provided a number of advancements to the previous environment and gene-regulatory influence network (EGRIN).[4] EGRIN models, learned by *cMonkey*[5,6] and *Inferelator*,[7] have accurately predicted conditional regulatory interactions in a number of organisms, including Mtb;[8–15] yet, these network models had some limitations in probabilistically uncovering conditional co-regulation of genes by specific TFs and GREs, especially in environmental conditions that represent only a small proportion of the input compendium of pathogen

Check for updates

This is an open access article under the CC BY license (http://creativecommons.org/licenses/by/4.0/).

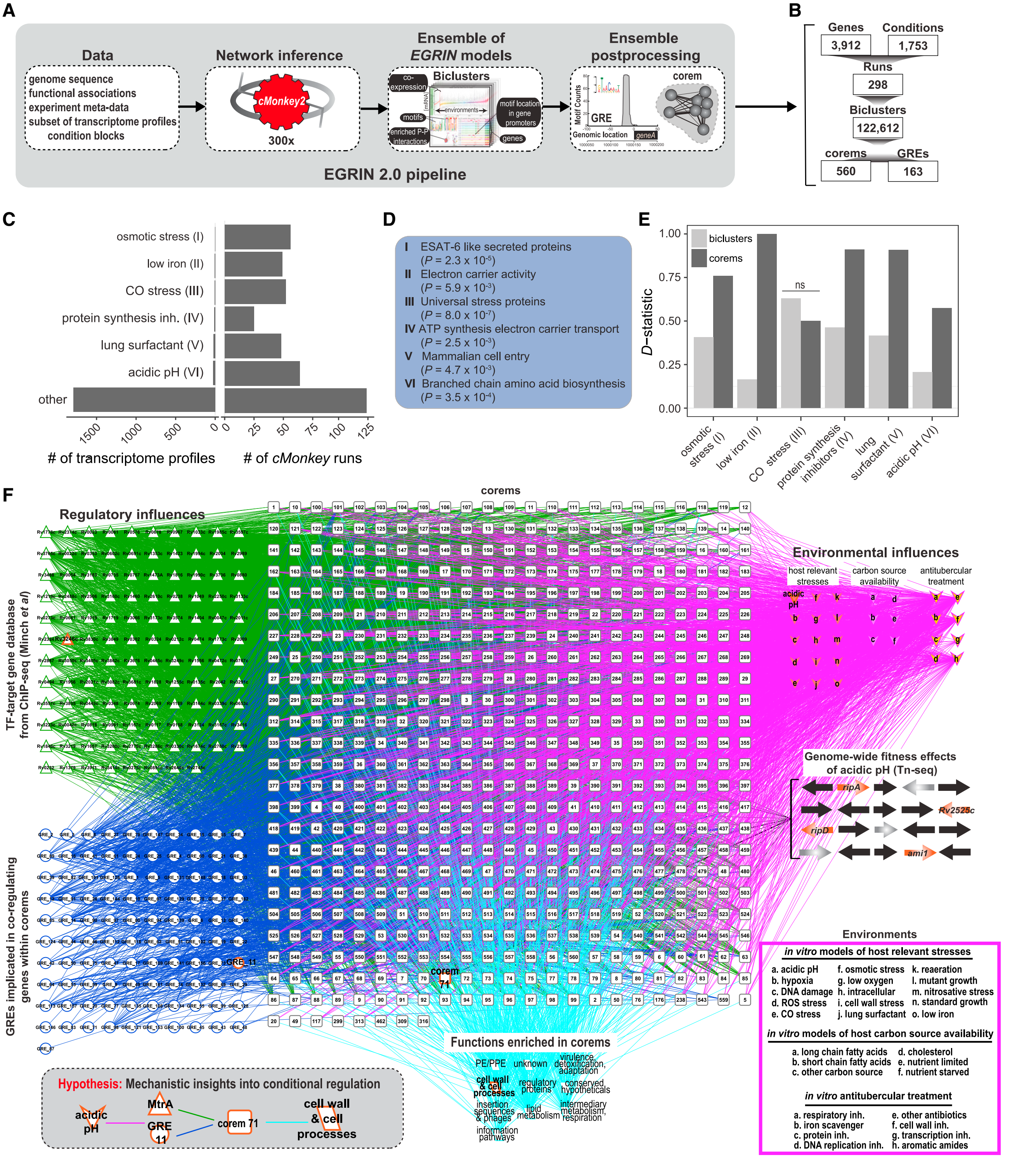

Figure 1. Overview of EGRIN 2.0 model for *M. tuberculosis*

(A) The *cMonkey* algorithm was applied many times to subsets of gene expression data from a large compendium of transcriptome profiles to construct many individual EGRIN models. The *cMonkey* biclustering algorithm identified sets of genes that have co-expression under a subset of experimental conditions, have a common motif in their promoters, and are enriched in protein-protein (P-P) interactions. Individual EGRIN models were integrated into an ensemble and mined to construct the final EGRIN 2.0 model, defining overlapping co-regulated sets of genes (corems) that are statistically associated with specific gene regulatory elements (GREs).

(legend continued on next page)

transcriptome profiles. Therefore, EGRIN 2.0 was developed to overcome these shortcomings and model the organization of GREs within every promoter and link the contexts in which they act to the conditional co-regulation of genes. Importantly, the improvements and algorithmic components of EGRIN 2.0 have been rigorously tested and validated in prior studies; readers are encouraged to refer to the original papers for more detail. As such, this study was able to focus on revealing new insights into the regulatory networks and physiological adaptations that Mtb undergoes over the course of infection. Here, we present an EGRIN 2.0 model of Mtb, focused on the bacterium's response to acidic pH to accomplish two goals: (1) understand the response to a stress with which Mtb must cope to survive and (2) demonstrate that an ensemble modeling approach can boost discovery of influences acting in difficult-to-probe and therefore underrepresented environmental contexts across the transcriptome compendium. Mtb colonizes environments where the pH can fluctuate, depending on macrophage activation status[16,17] or spatiotemporal variation in granulomas (ranging from pH 5.0–7.2, with a median pH of 5.5).[18,19] Thus, changes in pH may serve as a cue regarding the local microenvironment to direct Mtb to regulate processes that optimize its physiology for a specific niche.[20] While PhoPR is a well-characterized two-component system (TCS) that is required for Mtb response to pH,[21–23] Δ*phoPR* mutants are still capable of responding to acidic pH and high chloride,[22] suggesting other regulators may play critical roles in controlling Mtb response to pH in other environmental contexts. Interestingly, Mtb in acidic pH conditions represented only a small portion of the entire gene expression dataset for Mtb (less than 2%), yet the ensemble learning approach of EGRIN 2.0 boosted signal to noise to uncover regulatory mechanisms associated with acidic pH in the full ensemble.

Specifically, we identified MtrA, which is part of the MtrAB TCS, as a global modulator of Mtb response to pH as well as other host-related environmental cues. Although MtrAB was the first TCS to be characterized in Mtb and is essential for Mtb survival,[24–27] it remains poorly understood. MtrA deletion has primarily been studied in *M. smegmatis* (Msm),[28] a non-pathogenic rapidly growing mycobacterium; however, MtrA is not essential in Msm, which suggests the system does not behave identically across mycobacterial species. Other studies evaluating the role of MtrAB in Mtb were limited to analyzing gene expression changes.[28–32] Importantly, the environmental cues that drive the MtrAB system to coordinate changes in gene expression and functional consequences have not been comprehensively evaluated. Therefore, EGRIN 2.0 offers a systems view to explore the many remaining questions about MtrA's role and the many stimuli that trigger its action in Mtb. Using the Mtb EGRIN 2.0 model, we provide a mechanistic model for the role of MtrAB in facilitating Mtb growth arrest in response to multiple, related environmental cues. To validate EGRIN 2.0 predictions, we characterize the perturbation of MtrA directly in Mtb and, in so doing, establish the high therapeutic potential of the essential regulator.

RESULTS

Constructing EGRIN 2.0 for Mtb

EGRIN 2.0 is an ensemble framework that aggregates associations across genes, GREs, and environments from many individual EGRIN models, each trained on a subset of gene expression data. The aggregated, post-processed ensemble of EGRIN models is referred to as EGRIN 2.0, which details the organization of GREs within every promoter and identifies conditionally co-regulated gene modules, known as corems. The Mtb EGRIN 2.0 model was constructed using the same workflow as the previous EGIRN 2.0 models (Figure 1A), with a few advancements: (1) network inference was done with the *cMonkey2* biclustering alogirthm,[6] (2) TF-target gene interactions with physical binding (chromatin immunoprecipitation sequencing [ChIP-seq] data)[33] were used to inform the set-enrichment scoring module in *cMonkey2*, and (3) condition blocks of Mtb transcriptome responses to related environmental cues were manually defined to subset the gene expression data (Data S1). The compendium of gene expression data used for training Mtb EGRIN 2.0 was collected from publicly available datasets and previously used to construct the Mtb EGRIN model.[9] Within the expression compendium, transcriptome profiles of Mtb cultured in conditions of osmotic stress, low iron, carbon monoxide (CO) stress, rifampicin treatment, exposure to lung surfactant, or low pH were represented in less than 2% of the 1,900 transcriptomes in the compendium (Figure 1B). However, all condition blocks, including these, were included in ∼20% of *cMonkey2* runs aggregated into EGRIN 2.0. Thus, the ensemble-based method of EGRIN 2.0 can balance the relative contributions of datasets and reveal context-dependent regulatory interactions, even for environmental contexts that occur infrequently in the data. We compared the detection of enriched gene sets (Figure 1C) from these rare conditions within biclusters inferred by Mtb EGRIN and corems identified by Mtb EGRIN 2.0. With the exception of CO stress, corems were significantly better at detecting the enriched gene sets from rare conditions (Figure 1D). It is possible that associations related to CO stress

(B) A summary of the counts for each feature in the EGRIN 2.0 model for Mtb.
(C) Condition blocks representing <2% of all transcriptome profiles and their inclusion in *cMonkey* runs.
(D) General theme of most significant functional cluster defined by DAVID[35] for each condition block shown in (C).
(E) Enrichment of functional clusters in (D) for biclusters (EGRIN models containing relevant condition block) and corems of the EGRIN 2.0 model, using one-tailed Kolmogorov-Smirnov (KS) test. We report the KS *D* statistic. ns, not significant.
(F) The EGRIN 2.0 model of Mtb with 560 corems, each of which is statistically associated with GREs (blue circles) or transcription factors (TFs) from the ChIP-seq study by Minch et al.[33] (green triangles). Corems are also statistically associated with environmental influences (pink arrowheads) and general mycobacterial functions (teal parallelograms) defined by MycoBrowser.[36] We highlight a signal path through the network that is discussed in detail (bold edges and nodes): genes with similar fitness at acidic pH are significantly enriched in corem 71, which is predicted to be regulated by MtrA and GRE 11 and implicated in processes related to the cell wall. The inset illustrates ways in which the network model can be used to make actionable predictions. The diagram was generated with Biotapestry.[37] CO, carbon monoxide; inh, inhibitor(s).

would have been better identified if the condition block were consistently grouped with low oxygen (O_2) and nitric oxide (NO) stress transcriptomes, as CO, O_2, and NO are sensed concurrently during Mtb infection.[34] Overall, Mtb EGRIN 2.0 better captures conditional co-regulation of genes from rare environments. Given that low pH conditions represent only a small portion of the gene expression data, constructing an EGRIN 2.0 model for Mtb was imperative for this study (Figure 1E).

Genome-wide fitness screening identifies a corem with a fitness defect at acidic pH

To identify the regulatory networks involved in Mtb adaptation to acidic pH, we utilized transposon mutagenesis coupled with next-generation sequencing (Tn-seq).[38] Transposon mutant libraries containing ∼100K individual transposon mutants were generated in Mtb. The libraries were cultured in either pH 7.0 or pH 5.6 medium for 3 days, at which point DNA from the surviving bacteria was isolated. Transposon gene junctions were amplified and sequenced to identify transposon insertion sites and calculate their representation in each condition.[39] We summarized the comparative fitness of every gene at acidic pH versus neutral pH (“delta fitness”), and the acidic pH corem fitness was calculated by averaging the delta fitness scores for genes in each corem (Data S2). This analysis revealed corem 71 as being significantly enriched with genes associated with decreased fitness at acidic pH (permuted $p < 0.001$). To assess the specificity of the corem fitness association, we performed another Tn-seq fitness screen in the presence and absence of the cell-wall-perturbing detergent sodium dodecyl sulfate (SDS). There was no significant change in the fitness of corem 71 in the presence of SDS (Figure S1A and Data S2).

Analysis of EGRIN 2.0 model uncovers a role for corem 71 genes in the Mtb response to multiple environmental cues

We previously demonstrated, in *E. coli* and *H. salinarum*, how corems group together genes that have highly correlated fitness effects across environments, but are regulated by different TFs.[3] The high similarity of their expression changes across multiple environments brings them together into the same corem. Therefore, we explored all the environments enriched in corem 71 containing genes with a fitness defect in acidic pH. Enrichment of environmental influences in corems was assessed statistically and reported as p values using the hypergeometric test with Benjamini-Hochberg (BH) multiple testing correction. In addition to acidic pH ($p = 1.7 \times 10^{-8}$), corem 71 was also significantly enriched in hypoxia stress ($p = 4.6 \times 10^{-16}$), starvation ($p = 5.4 \times 10^{-8}$), and the response to respiratory inhibitors ($p = 4.7 \times 10^{-4}$). This may indicate a nexus of these cues during infection, specific to a location or status of the immune response.[22,40] Interestingly, NO, which is intimately linked to both respiration inhibition and hypoxia,[41,42] was not significantly enriched in corem 71. This could be explained by the previously reported finding that other forms of regulation, including post-translational modification, allosteric regulation of enzymes, and protein degradation, may play a bigger role in driving Mtb response to NO than transcription.[43] Moreover, the connection between acidic pH and respiratory inhibitors, specifically, is interesting and may be explained by a common response to redox imbalance (i.e., accumulation of reduced co-factors such as NADH/NADPH) known to occur at acidic pH[20,44] and during bedaquiline treatment.[45] Thus, this analysis identified a role for corem 71 genes in Mtb response to not just pH, but also hypoxia, starvation, and redox stress. Furthermore, similar analysis of environmental features associated with all corems identified six other corems that were also significantly enriched with the same set of environmental influences, again, suggesting that these stresses are linked in a particular host microenvironment. Exploring the intersection of genes across these corems, we found that all contained a gene encoding the peptidoglycan hydrolase RipA (Figure S1B),[46] indicating that RipA may play a vital role in Mtb adaptation to these environmental cues. In addition to these corems, *ripA* was found in corem 532, with significant enrichment of conditions related to cholesterol utilization ($p = 2.6 \times 10^{-3}$). Along with *ripA*, corem 532 genes are upregulated during growth in cholesterol (Figure S1C) and contains members of the *mce4* operon that play an essential role in cholesterol transport.[47]

MtrA regulation of corem 71 genes

To find evidence of corem 71 regulation by specific TFs, we investigated the EGRIN 2.0-predicted organization of GREs in the promoters of corem 71 genes. EGRIN 2.0 predicts the frequency of GRE alignment to each genomic position, thus predicting the organization of GREs in gene promoters at nucleotide resolution. EGRIN 2.0 predicts that five GREs are significantly enriched in the promoters of corem 71 genes, with GRE 11 being the most significant (BH-corrected $p = 8.8 \times 10^{-11}$) (Figure 2A). We compared the motif of GRE 11 to motifs that were deciphered through analysis of ChIP-seq-mapped TF binding locations and found significant sequence similarity to the regulator MtrA.[28,33] Other GREs can be similarly mapped, such as GRE 4 with significant similarity to DosR (Figure 2A).[48] Furthermore, MtrA binding sites were located by ChIP-seq in the promoters of corem 71 genes with statistical significance (BH-corrected $p < 0.0001$) (Figure 2B). In addition to the genes with reduced fitness at low pH, corem 71 contains 46 genes including MtrA. Given that MtrA is essential in Mtb,[28,49,50] we used CRISPR interference (CRISPRi)[51] to knock down the expression of *mtrA* and characterize its potential role in the regulation of corem 71 genes. We designed five small guide RNAs (sgRNAs) targeting *mtrA* in Mtb that achieved varying levels of knockdown (Table S1).[51] Two of the Mtb CRISPRi strains (with sgRNA2 and sgRNA3) reached high-level *mtrA* mRNA repression upon the addition of anhydrotetracycline (ATc), compared with controls without ATc (Figure S2A). With ATc induction, we also observed very limited growth (Figure S2B) and severe cell aggregation in broth culture (Figure S2C). The other three CRISPRi strains (with sgRNA4, sgRNA5, and sgRNA6) achieved lesser degrees of *mtrA* knockdown (Figure S5A) and growth reduction upon ATc addition (Figure S2D). Using sgRNA2 and sgRNA3, which produced high-level knockdown of *mtrA*, we collected samples 4 days after ATc addition for gene expression profiling by RNA sequencing (RNA-seq). We performed differential expression analysis with respect to uninduced (−ATc) controls and confirmed that CRISPRi knockdown of *mtrA* repressed the expression of many corem 71 genes (Figure 2C and Data S3). A total of 18 corem 71 genes with GRE

CellPress
OPEN ACCESS

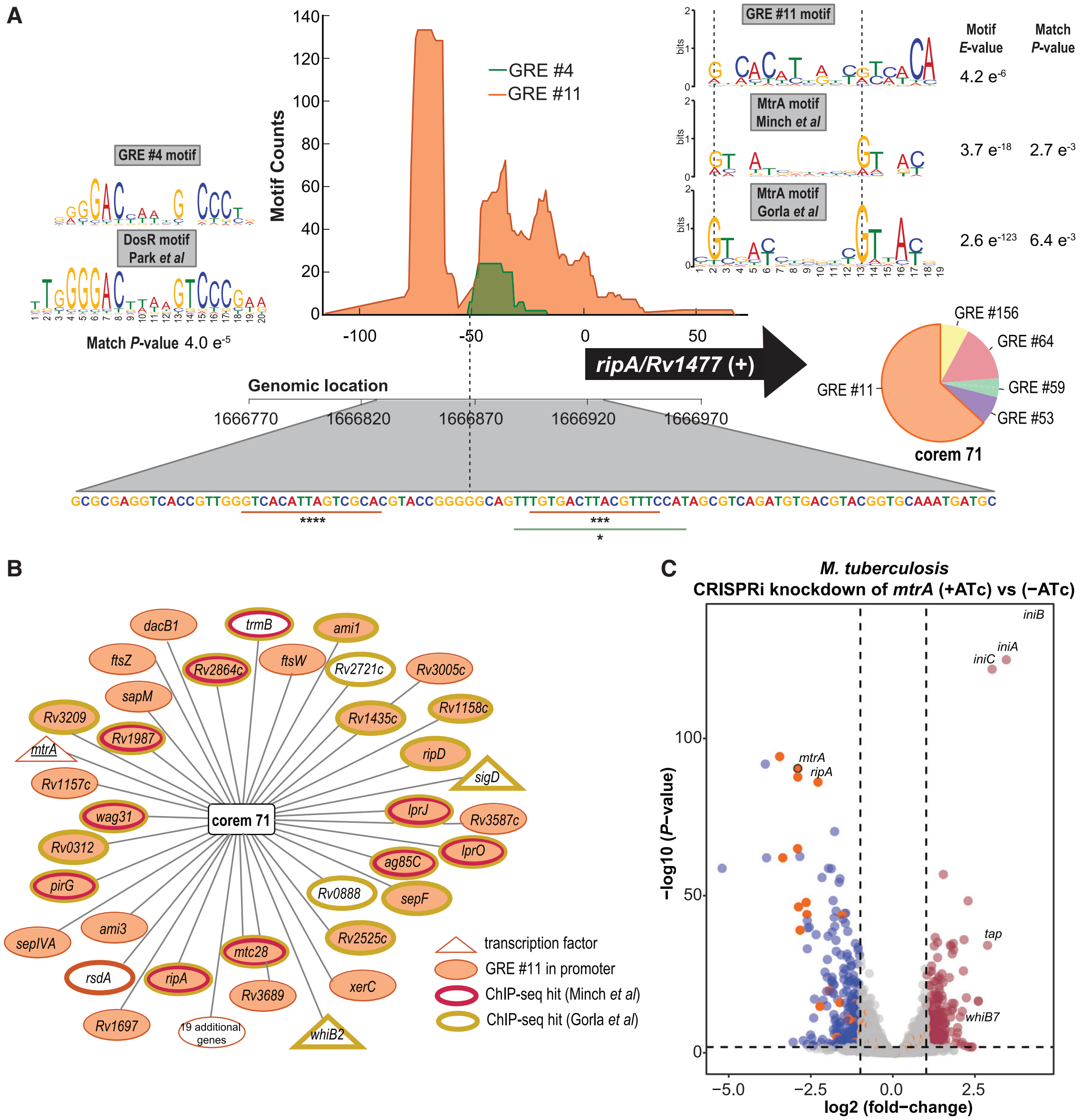

Figure 2. Validation of EGRIN 2.0-predicted regulation of corem 71 genes

(A) Architecture of the *ripA* (Rv1477) promoter predicted by the EGRIN 2.0 model. (Top) Frequency of GRE alignment to each position in the *ripA* promoter. Genome sequence marked with putative GRE 11 and GRE 4 locations with significance of alignment calculated by FIMO.[53] The motif logos of GRE 4 with DosR[48] and GRE 11 compared with MtrA motifs that were deciphered through analysis of ChIP-seq-mapped binding locations from Minch et al.[33] and Gorla et al.[28] are shown. The p values from alignment carried out with Tomtom[54] are shown. The pie chart represents average predicted influence of GREs on the regulation of genes in corem 71. *$p < 0.05$, ***$p < 0.001$, ****$p < 0.0001$.

(B) Network visualization of genes in corem 71.

(C) Volcano plot of differentially expressed genes for induced and uninduced CRISPRi knockdown of *mtrA* with sgRNA3 in Mtb. The significantly differentially expressed genes were selected by $p < 0.01$ and absolute log2 fold change >1. Dots represent different genes, with labels for particular genes of interest. Gray dots are genes without significant differential expression, red dots are significantly upregulated genes (n = 266 genes) and blue dots are significantly downregulated genes (n = 240 genes). The orange dots are all genes of corem 71.

11 in their promoter were significantly repressed upon *mtrA* knockdown (Table S2). A handful of other genes (*Rv1075c*, *lipU*, *ctpD*, *ripB*, *Rv1754c*, and *desA3*) were significantly downregulated with *mtrA* knockdown but not represented in the model and could also be *bona fide* regulatory targets of MtrA. Further, the isoniazid-induced genes (*iniBAC*) were significantly upregulated when *mtrA* expression was silenced (Figure 2C), a well-known indicator of defects in the cell wall.[52]

Given the strong homology (84.1% nucleotide sequence similarity and 96.9% amino acid identity) but difference in essentiality

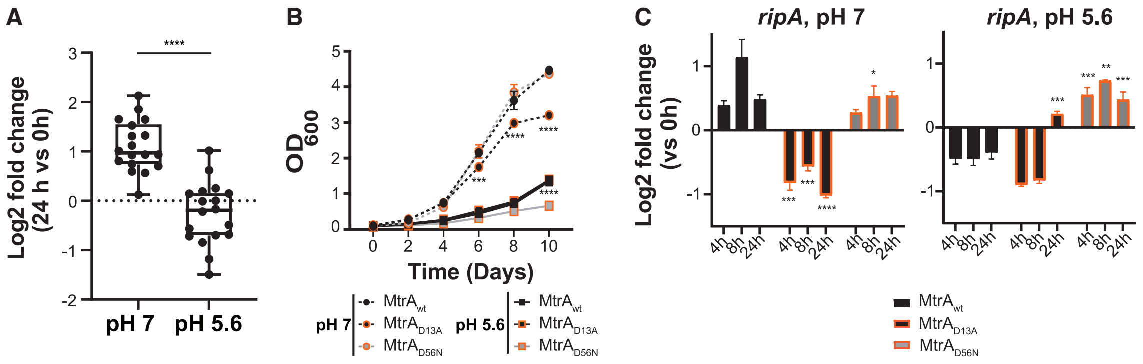

Figure 3. Characterization of MtrA activity in acidic pH

(A) Boxplot representing log2 fold change for Mtb H37Rv in medium buffered to either pH 7 or pH 5.6, comparing expression at 24 h versus 0 h of MtrA regulatory targets in corem 71 (unshaded genes in Table S2) from three biological samples.

(B) Growth of Mtb indicated merodiploid strains producing wild-type MtrA or MtrA mutants in medium buffered to either pH 7 (circles, dashed line) or pH 5.6 (squares, solid line). Growth was monitored by optical density at 600 nm. Points are the means of three biological replicates and error bars represent standard deviation (SD).

(C) Bar plot representing log2 fold change of *ripA* from Mtb merodiploid strains in neutral or acidic pH, comparing expression at 24 h versus 0 h. Data are the mean ± SD from three biological samples. Statistical significance was calculated with Student's t test. *$p < 0.05$, **$p < 0.01$, ***$p < 0.001$, ****$p < 0.0001$.

of MtrA in Mtb and Msm,[28,55] we sought to also characterize *mtrA* knockdown in Msm. We generated CRISPRi strains that achieved strong repression of *mtrA* upon ATc addition (Figure S3A), but resulted in only moderate growth inhibition of Msm (Figure S3B), supporting the prediction that MtrA is not essential in Msm, in contrast with the Mtb ortholog. We performed RNA-seq and analyzed differential expression of all genes due to ATc-induced repression of *mtrA* in the Msm CRISPRi strain (Figure S3C and Data S4). Knockdown of *mtrA* in Msm resulted in significant repression of many genes orthologous to the MtrA regulon in Mtb (Table S2) and a consensus binding motif with significant overlap to GRE 11 and MtrA from ChIP-seq (Figure S3D). There were also key differences, of note, two genes with conserved DivIVA domains, *wag31* (*MSMEG_4217*) and *sepIVA* (*MSMEG_2416*), that are essential for cell growth in both species.[56,57] Both genes were repressed upon *mtrA* knockdown in Mtb, but *wag31* was not differentially expressed in Msm (and no MtrA consensus motif found in promoter), while *sepIVA* was significantly upregulated and had a strong MtrA motif found in its promoter ($p = 3.7 \times 10^{-5}$). It is possible that changes in interaction with these essential target genes could explain the difference in MtrA essentiality between Msm and Mtb. In addition, repression of *mtrA* in Msm caused significant upregulation of genes encoding the peptidoglycan (PG) transglycosylases known as resuscitation promoting factors, *rpfB* and *rpfE* (log2 fold change = 2.47 and 5.11, respectively, $p < 0.01$). RpfB and RpfE interact with RipA (Rpf-interacting protein) in Msm, where the protein complex hydrolyzes PGs during cell division.[58–60] It is possible that increased expression of the *rpfs* (via an unknown regulatory mechanism) could sustain PG cleavage in Msm when other PG hydrolases (*ami1*, *ripA*, *ripD*) were repressed by *mtrA* silencing, making *mtrA* dispensable for growth in Msm. In Mtb, *rpfB* and *rpfE* were not significantly differentially expressed upon *mtrA* knockdown, which points toward differences in PG remodeling pathways between the two species.[58,61,62] This also suggests that, while the regulatory function of MtrA is largely conserved from Msm to Mtb, differences in the activity of the MtrA regulon have emerged upon their evolutionary divergence, manifesting as a particular vulnerability for the human pathogen.

MtrA regulon repression slows Mtb growth in response to environmental cues

To examine the transcriptional response of the MtrA regulon at low pH, we grew wild-type Mtb to mid-log phase in 7H9 growth medium at neutral pH and washed and then diluted the cells in either neutral or low-pH medium (7H9-rich medium supplemented with 0.05% tyloxapol and adjusted to pH 7.0 or 5.6). Following transition to either neutral or acidic medium, we monitored growth for 7 days and collected samples at 24 h for transcriptome profiling by RNA-seq. We observed a significant decrease in MtrA regulon expression at acidic pH compared with neutral pH (Figure 3A), along with an expected decrease in Mtb growth at acidic pH (Figure 3B). This change in expression of MtrA regulon genes suggests that an appropriate reduction in their expression is important for proper growth (i.e., growth arrest) in acidic conditions. This could explain why transposon mutants with cell growth defects were more sensitive to acid stress. We also see significantly decreased expression of the MtrA regulatory target, *ripA*, in response to the conditions enriched in corem 71 (hypoxia, starvation, respiratory inhibitors), but, strikingly, no significant transcriptional response to NO stress (Figure S4), consistent with model predictions. Altogether, this evidence points toward MtrA repression of an ~25 gene regulon, which slows growth and promotes survival in response to multiple environmental stresses.

To test whether perturbed MtrA activity would alter Mtb response to acid stress, we used two MtrA phosphorylation-deficient mutants. One mutant had alanine in place of aspartic acid at the 13th codon ($MtrA_{D13A}$)[63] and the other had the aspartic acid at codon 56 replaced with asparagine ($MtrA_{D56N}$).[30] Al Zayer et al. previously demonstrated that the merodiploid strain producing $MtrA_{D13A}$ grew poorly in nutrient-rich broth, suggesting

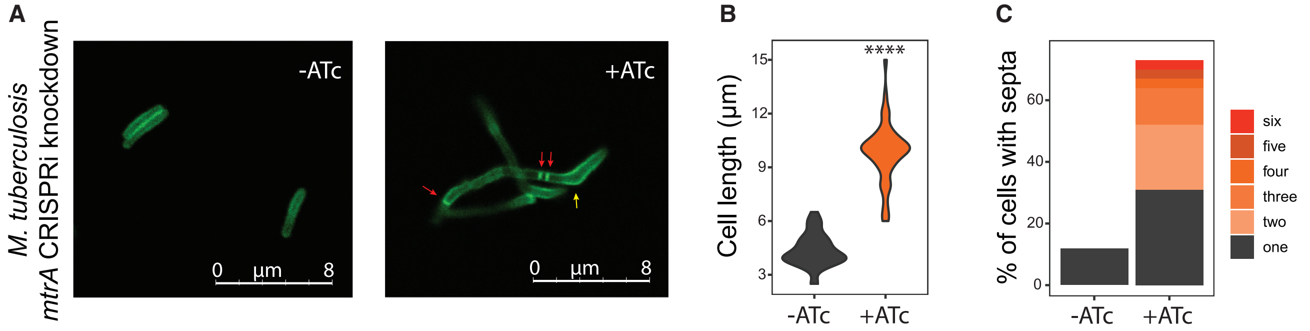

Figure 4. MtrA controls cell division in *M. tuberculosis*

(A) Example micrographs of uninduced (−ATc) and induced (+ATc) CRISPRi knockdown of *mtrA* with sgRNA5 in Mtb. After knockdown, cells were labeled with HCC-amino-D-alanine (HADA) for 20 h. Red arrows indicate multiple septa and yellow arrow indicates the polar clubbing phenotype.

(B) Violin plots showing the cell length of uninduced and induced CRISPRi knockdown of *mtrA* with sgRNA5 in Mtb. Significance was determined by unpaired t test. ****$p < 0.0001$.

(C) Bar plot representing the percentage of cells that contain one or more septa for uninduced and induced CRISPRi *mtrA* knockdown with sgRNA5 in Mtb. The colored sections within the bars represent the proportions of cells with different numbers of septa. Two independent experiments were quantified. ATc, anhydrotetracycline inducer.

that $MtrA_{D13A}$ behaves like a constitutively active repressor.[63] Consistent with these results, we observed a growth defect for $MtrA_{D13A}$ at neutral pH (Figure 3B). However, there was no growth defect compared with wild-type MtrA ($MtrA_{wt}$) at acidic pH, and we found similar repression of the MtrA regulatory target, *ripA*, at both neutral and acidic pH (Figure 3C). Previous characterization showed that the merodiploid strain producing $MtrA_{D56N}$ had no growth defect in standard broth conditions, but that the mutant was only partially replicative in macrophages compared with wild-type Mtb and was attenuated in mice.[30] We also found no growth defect with $MtrA_{D56N}$ at pH 7, but observed a growth defect at acidic pH compared with $MtrA_{wt}$ (Figure 3B). Gene expression analysis also revealed significant upregulation of *ripA* at acidic pH, which is in contrast to the downregulated expression in $MtrA_{wt}$ during acid stress (Figure 3C). Thus, alterations in MtrA phosphorylation status at specific residues can disrupt MtrA interaction with its regulatory target, making Mtb maladaptive to conditions of active replication (i.e., $MtrA_{D13A}$ at neutral pH) and slow growth (i.e., $MtrA_{D56N}$ at acidic pH). While more studies are needed to determine the exact phosphorylation status of MtrA in actively growing versus slowly growing cells (and in response to particular stress conditions), these results confirm that precise MtrA phosphorylation is required for optimal MtrA target expression in response to environmental cues.

Knockdown of *mtrA* results in cell division and elongation defects

Among the proposed regulatory targets of MtrA (Table S2) are five genes that encode proteins with PG hydrolase activity: *ami1*, *ripA*, *ripB*, *ripD*, and *Rv2525c*. PG hydrolases are present in multiple variant forms in all bacteria, where their activities are harnessed to support cell growth, division, and differentiation, enabling bacteria to propagate and adapt to changing environmental conditions.[64,65] The enzymes (PG hydrolases) have major roles in cleavage of the septum during cell division and breakdown of PGs to accommodate new material during cell elongation.[64] PGs consist of linear glycan strands cross-linked by short peptides to form a rigid polysaccharide layer between the outer "mycomembrane" and the plasma membrane. A variety of hydrolase enzymes allow for the specific cleavage of different positions on the macromolecule, and the required hydrolase activity may be specific for certain processes and/or conditions.[66] Among the multiple PG hydrolase targets of MtrA, Ami1 contains an amidase_3 domain that cleaves the peptide stem from N-acetyl-muramic acid on the PG glycan backbone.[67] RipA and RipB are D,L-endopeptidases that cleave PGs within the peptide stem.[68] RipD also contains an endopeptidase NlpC/p60 domain, similar to RipA, but the purified protein demonstrated a non-catalytic PG-binding function.[69] Finally, structural studies suggest a PG glycoside hydrolase function for Rv2525c, although its activity has not been established.[70] To assess the effect of MtrA regulation on these PG cleavage enzymes and the impact on Mtb cell division and growth, we used the HCC-amino-D-alanine analog (HADA), which is incorporated into newly synthesized PGs.[71] We fluorescently labeled the PG of Mtb *mtrA* knockdown cells containing sgRNA5, which achieved low-level *mtrA* repression, and observed elongated multiseptated cells (Figure 4A). Cells with ATc-induced *mtrA* knockdown had an increased median cell length compared with uninduced cells (Figure 4B). Nearly all (~75%) of the *mtrA* knockdown cells contained at least one septum, while septa were present in only 15% of the uninduced cells (Figure 4C). In fact, more than one septum was present in a majority of the *mtrA* knockdown cells, indicating that septa form, but the cells fail to divide, and MtrA regulatory targets act at late stages of cell division. In addition, the *mtrA* knockdown cells adopted an abnormal clubbed or bent shape at the poles, which was not found in the uninduced cells (Figure 4A). Interestingly, one pole was affected more than the other in most cells, and fluorescent signal was greater in the region of the pole that was clubbed (Figure 4A). The unipolar clubbing and HADA accumulation evident in *mtrA* knockdown cells suggest that MtrA regulatory targets may also play a role in the incorporation of new PG material during elongation, which occurs at the poles in mycobacteria.[72] This unipolar phenotype is consistent with irregular

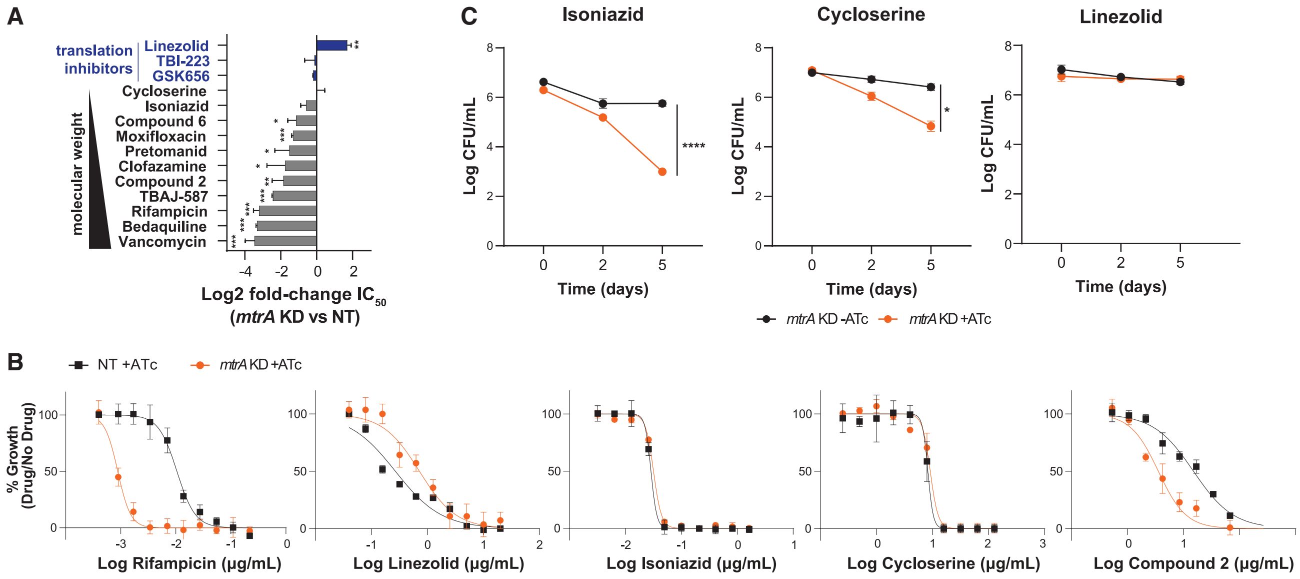

Figure 5. MtrA promotes intrinsic resistance and drug tolerance

(A) Bar plot representing the log2 fold change in IC_{50} for the indicated drugs in the *mtrA* knockdown stain compared with the control. The gradient of molecular weights is also indicated. Results from an unpaired t test are shown; ***p < 0.001, **p < 0.01, *p < 0.05.

(B) Dose-response curves for the indicated strains. Data are means ± SD from three biological samples and representative of two independent experiments.

(C) Colony-forming unit (CFU) quantification of the indicated strains after incubation with isoniazid (0.25 μg/mL), cycloserine (15 μg/mL), or linezolid (2.0 μg/mL) in standard growth medium. Data are means ± SEM from two independent experiments, each performed with triplicate cultures. Statistical significance of the differences between control and *mtrA* knockdown was assessed by unpaired t test, *p < 0.05, ****p < 0.0001. NT, non-targeting sgRNA; KD, knockdown; ATc, anhydrotetracycline inducer.

localization of new growth in mycobacteria, specifically caused by dysfunction in Wag31 due to a fluorescent protein tag.[73] Thus, the observed phenotype reveals that MtrA might not be required only for septal PG cleavage during cell division, but may also facilitate cell growth at the polar region via regulation of *wag31*. Elongated, multiseptated cells, exhibiting polar clubbing, were also observed with the Msm *mtrA* knockdown strain, although the fluorescent signal was more uniform (Figure S5).

MtrA promotes intrinsic drug resistance to high-molecular-weight antibiotics

In a genome-wide CRISPRi library screen against a panel of diverse antibiotics, *mtrA* knockdown strongly sensitized Mtb to rifampicin, vancomycin, and bedaquiline, mediated by increased cell-wall permeability.[32] Independently, we measured the IC_{50} (concentration required for 50% growth inhibition) using the *mtrA* CRISPRi strain with sgRNA5. Consistent with the screening results, *mtrA* knockdown decreased the Mtb IC_{50} to vancomycin, bedaquiline, and rifampicin, but not isoniazid (Figures 5A and 5B). Also consistent with the screening results, there was decreased susceptibility to the translation inhibitor linezolid. Extending these results, we quantified drug susceptibility of *mtrA* knockdown with additional antitubercular compounds, including two compounds reported as MtrA inhibitors (compound 2 and compound 6).[74] With the exception of translation inhibitors, the IC_{50} reduction (calculated as log2 fold change for *mtrA* knockdown compared with control) was significantly correlated with the molecular weight of the compounds tested (R^2 = 0.68), more than other drug physiochemical properties (Figure S6A). There were also similar molecular-weight-dependent drug susceptibility patterns observed in *mtrA* knockdown cultures in Msm, as measured by disk diffusion (Figure S6B). The Msm *mtrA* knockdown cultures showed increased susceptibility to rifampicin, carbenicillin, and vancomycin, but no difference in susceptibility for cycloserine and isoniazid, compared with the control.

The reported MtrA inhibitors, compound 2 (C2) and compound 6 (C6), both based on a thiazolidine scaffold, had wild-type Mtb IC_{50} measurements of 15 and 350 μg/mL, respectively, higher than previously published (9 and 43 μg/mL, respectively).[74] However, *mtrA* knockdown decreased the IC_{50} 3-fold for both C2 and C6. Further, we looked at gene expression changes in wild-type Mtb treated for 72 h with C2 (34 μg/mL) or C6 (80 μg/mL) compared with DMSO control. Treatment with C2 significantly upregulated the MtrA regulon, whereas C6 did not have any effect on the expression of MtrA regulatory targets (Figure S6C). While we cannot conclusively determine that these compounds directly exert effects on MtrA (although hypersensitivity of hypomorphs is well established in Mtb[75–77]), these results demonstrate how inhibition of MtrA can potentiate antitubercular compounds, particularly inhibitors of new targets, which are desperately needed to treat TB.

MtrA promotes drug tolerance to cell-wall-targeting antibiotics

Mycobacteria divide asymmetrically, resulting in daughter cells that have different growth rates, sizes, and distributions of macromolecules.[72] One daughter cell, called the alternator cell, inherits one non-growing pole of intermediate age and one new pole. Its sister cell, the accelerator cell, inherits a new pole and

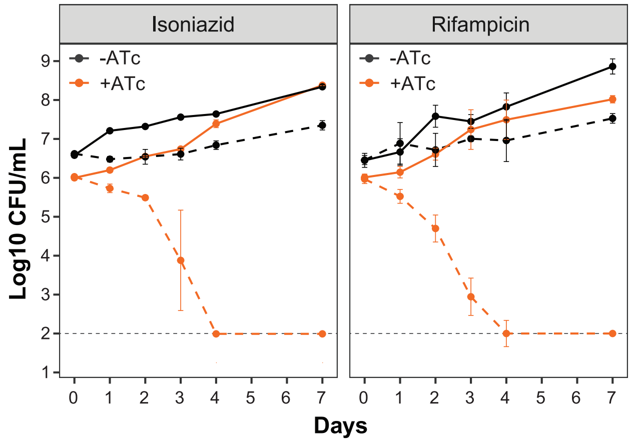

Figure 6. The knockdown of *mtrA* potentiates frontline TB drugs
Colony-forming units (CFU) of Mtb over a period of 7 days for untreated (solid lines) and 0.018 μg/mL isoniazid or 0.006 μg/mL rifampicin-treated (dotted lines) liquid cultures of uninduced (black, minus ATc) and induced (orange, plus ATc) CRISPRi knockdown of *mtrA* with sgRNA5. Error bars show the standard deviation from three biological samples and gray dotted lines represent the limit of detection. Representative results from two experiments are presented. ATc, anhydrotetracycline inducer.

the oldest pole and elongates faster from the old pole. The faster elongation (and possibly cell-wall differences) makes accelerator cells more susceptible to cell-wall synthesis inhibitors. Because *mtrA* knockdown cells were elongated and exhibited polarized cell-wall accumulation, we hypothesized that the population may more resemble accelerator cells and be killed more rapidly by drugs that target the cell wall. Indeed, the kinetics of isoniazid- and cycloserine-mediated killing in liquid cultures were faster for the *mtrA* knockdown than for the control Mtb (Figure 5C). As the IC_{50} measurements of isoniazid and cycloserine were similar for the control and *mtrA* knockdown, these results indicate that MtrA regulatory activity confers tolerance to isoniazid and cycloserine. Knockdown of *mtrA* did not enhance the killing by linezolid, which also exhibited no difference in IC_{50} between *mtrA* knockdown and control, suggesting that tolerance mediated by MtrA may be specific to cell-wall-targeting antibiotics.

MtrA knockdown potentiates frontline antibiotics

The penetration of antibiotics into the Mtb lesion can be limited, causing Mtb to be exposed to fluctuating and often subinhibitory concentrations of antibiotic. We compared time-kill curves for the induced *mtrA* knockdown strain with the uninduced strain when exposed to low (below IC_{50}) concentrations of isoniazid or rifampicin. The two drugs have different mechanisms of action and different mechanisms by which MtrA confers survival, yet at concentrations that only partially inhibited growth in the uninduced controls, no culturable bacteria were detected in the *mtrA* knockdown samples after 4 days of treatment (Figure 6).

DISCUSSION

The EGRIN 2.0 model for Mtb reveals how the pathogen tailors its transcriptional responses to survive disparate stressors in the highly distinctive microenvironments experienced within the host. Merging an ensemble of EGRIN models, each tuned to a relatively smaller subset of the overall input transcriptome compendium,[3] allows EGRIN 2.0 to mechanistically distinguish, among otherwise highly similar responses, important nuances that occur in rare contexts. Here, we demonstrated that ensemble modeling greatly improved detection of regulatory mechanisms that are active in conditions that represent only a small proportion of the entire dataset, such as acidic pH, a stress that Mtb must cope with to survive. We identified a regulatory module (corem 71) that brought together genes with similar fitness contributions in acidic pH and significant co-expression across diverse intracellular environmental contexts, including acidic pH, hypoxia, starvation, and response to respiration inhibitors. Together, these different stresses may represent a specific niche within the host microenvironment, and corem 71, a transcriptional circuit that modulates key functions to support Mtb survival therein. Such environmental associations would be difficult to make with *in vitro* studies of Mtb response to environmental cues that largely focus on one (maybe two) signal at a time. While *in vivo* spatially resolved transcriptomic profiling[78] of Mtb within infected tissues (e.g., lung granuloma) from animal models and, when accessible, directly from humans will aid in discovery of such context-specific regulatory mechanisms, it would be challenging to discern the specific environmental drivers of the response. Instead, EGRIN 2.0 can partly resolve these limitations by integrating and resampling transcriptomes from a broad range of relevant conditions and mining for correlated expression patterns and associated GREs observed reproducibly in a context-dependent manner. The discovery of regulatory mechanisms that operate across contexts is feasible because the architecture of regulatory networks within microbes reflects an internalized representation of the physicochemical coupling between environmental signals in relevant niches they occupy (e.g., low O_2, nutrient starvation, and acidic pH within a granuloma). Hence, perturbation in one environmental factor often elicits a response that is similar to that elicited by other co-occurring environmental factors.[79,80] Thus, EGRIN 2.0 can provide insight into coordinated changes in multiple environmental factors experienced by Mtb in a host microenvironment and how it adapts to a given niche.

EGRIN 2.0 predicts that GRE 11 is putatively responsible for mediating transcriptional regulation of many corem 71 genes. The locations of GRE 11 align with regions that were experimentally characterized in independent studies as MtrA binding sites, demonstrating that EGRIN 2.0 is able to accurately predict the organization of GREs in gene promoters at nucleotide resolution. Strikingly, we found that GRE 11 was active in environments, including acidic pH, where the MtrA-regulated transcript, *ripA*, is repressed, but also in environments, including growth on cholesterol, where transcript levels are elevated. This is powerful because it shows that, using EGRIN 2.0, we can predict when (environmental context) and how (activate or repress) a specific GRE within a promoter might act, even though we might not know the precise regulatory mechanism (e.g., TF binding/unbinding, post-translational modification, co-factor interaction, etc.). Furthermore, these alternate promoter states correspond to co-regulation of *ripA* with a different combination of genes

(i.e., different corems). Thus, the context in which GRE 11 is active may point to distinct functions of RipA. Indeed, recent work established the contextual redundancy of RipA and Ami1, both PG cleavage enzymes and regulatory targets of MtrA, by establishing that Ami1 sustains *in vitro* cell division in Mtb cells lacking *ripA*, but that this redundancy is not sufficient for replication and persistence of Mtb *in vivo.*[81] Thus, RipA activity may be associated with specific conditional or temporal events during infection, possibly related to host-derived cholesterol utilization, as predicted by EGRIN 2.0.

We observed that MtrA controls ~30 genes and reduces their expression under growth-compromising conditions. In particular, we reveal that the MtrA regulon includes a number of PG cleavage enzymes (*ripA*, *ami1*, *ripB*, *ripD*, and *Rv2525c*). Interestingly, corem 71 contains three other PG hydrolases, *dacB1* (Rv3330), penicillin-binding lipoprotein (Rv2864c), and *ami3* (Rv3811), that also have a dominant GRE 11 found in their promoter region and were mildly but significantly upregulated with *mtrA* knockdown in Mtb ($p < 0.01$). PG hydrolases form a vast group of enzymes (glycosidases, amidases, endopeptidases, and carboxypeptidases), with multiple homologs of each in Mtb. They have the selective ability to cleave PGs and play a central role in many processes (e.g., cell growth, cell division, resuscitation),[82] but are capable of lysing whole cells when dysregulated. Therefore, regulation of multiple PG hydrolases by the same TF (i.e., MtrA) may ensure that PG degradation is coordinated across most environments (i.e., low pH, hypoxia, starvation), but also subject to fine-tuned changes in some contexts by the dual phosphorylation of MtrA, TFs binding to other GREs in the gene promoters (e.g., GRE 4/DosR in *ripA* promoter), and activation by other proteins (e.g., MarP cleavage of RipA and CwlM interaction with MurA).[46,83]

Several recent studies have evaluated MtrA regulatory targets using global ChIP-seq analysis with Mtb strains overexpressing C-terminal FLAG-MtrA,[33] N-terminal His-tagged MtrA,[84] or proposed phosphorylation-competent MtrA protein (replacement of tyrosine with cysteine at position 102).[28] Interestingly, none of the 14 putative MtrA targets commonly identified across these studies had GRE 11 identified in their promoter. Only *Rv1815* and *rpfC* were found together in corems (with same conditional enrichment as corem 71) and were significantly differentially expressed with Mtb *mtrA* knockdown (log2 fold change = −1.9 and −2.5, respectively). The other genes consistently associated with MtrA binding across all the studies were not represented in EGRIN 2.0, which might suggest their co-regulation in a context that was not represented in the transcriptome compendium or a limitation in coverage of the current model. Such shortcomings could be addressed by expanding the ensemble with a larger number of EGRIN models, generated with a greater diversity of transcriptome profiles from infection-relevant contexts in which important, albeit rare, regulatory events may occur. In particular, high-resolution longitudinal gene expression profiling during environmental transitions[85] may capture intermediate adaptive processes that are not included in the current model.

In a typical TCS, the membrane-embedded histidine kinase responds to environmental signals by either phosphorylating or dephosphorylating a cognate response regulator, usually on an aspartate residue.[86,87] The DNA binding activity of the response regulator is thereby altered because of changes in its phosphorylation status. In this study, we showed that Mtb merodiploid strains producing elevated levels of phosphorylation-deficient $MtrA_{D13A}$ or $MtrA_{D56N}$ were attenuated for growth in broth at neutral or acidic pH, respectively. Further, we found that mutation of specific aspartate residues led to perturbed expression of the target gene, *ripA*, thereby conditionally interfering with growth. Together, these data indicate that the combination of D13 and D56 phosphorylation enables the integration of environmental signals to fine-tune MtrA-mediated gene regulation and growth of Mtb. It is pertinent to note that *mtrB* is not essential for growth, indicating phosphorylation-independent regulation[29] and/or that other kinases phosphorylate MtrA.[24] Recently, it was shown that MtrA DNA binding activity increased when phosphorylated by PknK,[88] whereas PknA/B-mediated MtrA phosphorylation decreased its DNA binding ability.[89] Therefore, there is a distinct possibility of a complex combinatorial scheme with multiple kinases phosphorylating multiple sites on MtrA, with differential consequences on its function as both an activator and a repressor.[30] This scheme is reminiscent of other response regulators with dual phosphorylation sites, such as the virulence-related response regulator CovR of group A *Streptococcus* (also within the OmpR/PhoB family of response regulators), which has competing phosphorylation sites as well as a phosphorylation-independent mechanism of gene activation.[90] Altogether, it is likely that the phosphorylation status of MtrA is influenced by a multitude of factors and, by integrating these layers of post-transcriptional regulation into EGRIN 2.0, we could elucidate how such phosphorylation ultimately influences gene expression and generates a more complete understanding of Mtb pathogenesis.

Using EGRIN 2.0 and CRISPRi to repress *mtrA* in Mtb, we have identified a number of regulatory targets for the essential response regulator (Table S2). Moreover, we have conducted parallel experiments in Msm and found conserved MtrA binding motifs and regulatory targets between the species. However, there were also key differences (e.g., *wag31*, *sepIVA*) that may point to why *mtrA* is dispensable for growth in Msm but not Mtb. These results call into question the relevance of using a fast-growing mycobacterial model organism to interpret MtrAB activity in Mtb. This caution may extend to the study of mycobacterial PG-remodeling enzymes in general, as there are fundamental differences in PG synthesis and breakdown between Msm and Mtb.[81,91–93] Further, our findings suggest that evolution from saprophyte to pathogen likely occurred, in part, through reprogramming of the MtrAB system, through alterations in (1) dynamics/context of MtrA phosphorylation, (2) its TF-TF and promoter interactions, and (3) the target genes in its network, to collectively afford Mtb better adaptation to the nuances of the host environment.[94,95]

Our findings also showed that *mtrA* knockdown in Mtb strongly influences intrinsic resistance to rifampicin, vancomycin, and bedaquiline[32] and renders Mtb more susceptible to all drugs tested with molecular weights above 200 g/mol (including compounds with unknown mechanisms of action), but with no altered sensitivity to translation inhibitors. It seems likely that MtrA confers intrinsic drug resistance by regulating genes that contribute to the permeability barrier of the cell wall, thereby

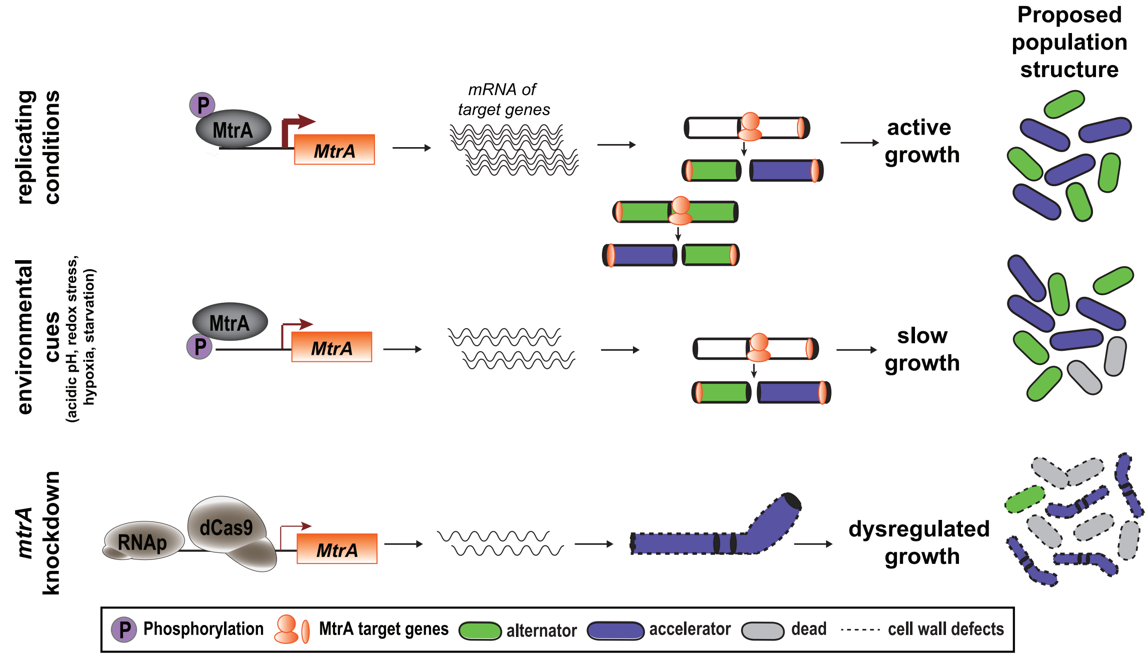

Figure 7. MtrA regulation of Mtb growth and antibiotic susceptibility

Under nutrient-rich conditions, MtrA binds to the promoter and induces the expression of a number of target genes that are important for cell division and growth as well as contributing to the cell wall as a barrier to antibiotics. Asymmetric cell division in mycobacteria promotes a heterogeneous bacteria population that allows some bacteria to survive TB therapy. In response to environmental cues, a change in the phosphorylation status of MtrA relieves binding and expression of target genes, resulting in a slow-growing but stillheterogeneous population. Knockdown of *mtrA* enhances the permeability of antibiotics into the cell, thereby increasing the sensitivity of Mtb to antibiotics with molecular weight greater than 200 g/mol (excluding translation-targeting antibiotics). In addition, knockdown of *mtrA* leads to dysregulated division and growth that makes the population more sensitive to cell-wall inhibitors. We speculate this is due to loss of heterogeneity and/or enrichment of alternator-like cells.

restricting access of high-molecular-weight antibiotics to their intracellular or periplasmic targets. Isoniazid and cycloserine are low-molecular-weight molecules (under 200 g/mol) and are therefore able to pass through the cell wall equally well in the *mtrA* knockdown and wild-type strains. Interestingly, the molecular weight cutoff does not apply to translation-targeting drugs, as *mtrA* knockdown did not sensitize Mtb to three protein synthesis inhibitors, TBI223, GSK3036656, and linezolid, despite their molecular weights being above 200 g/mol. We speculate that these compounds also have greater uptake in the *mtrA* knockdown strain, but that slower growth (in environmental conditions and especially upon *mtrA* knockdown) may result in repressed activity of their protein synthesis targets.[96] We also observed significant upregulation of the drug efflux pump *tap* and its TF, *whiB7*, with *mtrA* knockdown (Figure 2). Expression of the *whiB7* regulon induces a stress response that promotes intrinsic resistance to numerous ribosome-targeting antibiotics.[32,97,98] It would be interesting to further investigate how MtrA regulation affects translation and/or WhiB7 expression and thereby the activity of protein synthesis inhibitors.

Knockdown of *mtrA* resulted in faster rate of killing with the cell-wall inhibitors isoniazid and cycloserine, with no change in IC_{50} measurements, indicating that MtrA mediates tolerance and not resistance to these drugs. We speculate that genes regulated by MtrA (e.g., *wag31*, *sepIVA*) are important for asymmetric cell division in Mtb, which can lead to a heterogeneous population with individuals that have different drug sensitivity (Figure 7). Drug-tolerant subpopulations are of particular interest as they are thought to contribute to the unusually long TB treatment, and targeting these subpopulations has the potential to shorten TB chemotherapy. Therefore, further work at the single-cell level is needed to determine whether MtrA contributes to a heterogeneous population of cells with variation in drug susceptibility. Here, we have reported on a single protein as a mediator of both intrinsic resistance and drug tolerance in Mtb. Its essentiality for growth (both extracellular and intracellular) and ability to potentiate diverse drugs make MtrA a promising drug target, illustrating the power of EGRIN 2.0 in facilitating discovery of novel targets that operate in specific host microenvironments.

Limitations of the study

EGRIN 2.0 facilitates the discovery of conditional genome-encoded regulatory programs in Mtb and is useful for both low- and high-profile environmental contexts, including those that represent only a small proportion of the input compendium of transcriptomes. The ensemble-based method of EGRIN 2.0 can balance the relative contribution of datasets from various conditions. However, this procedure is ineffective for contexts that are absent from the transcriptome compendium and could explain a limitation in coverage of the current EGRIN 2.0 model of Mtb. Such shortcomings could be addressed by expanding

the ensemble with a larger number of EGRIN models generated with a greater diversity of transcriptome profiles from infection-relevant contexts in which important, albeit rare, regulatory events may occur. In particular, high-resolution longitudinal gene expression profiling during environmental transitions may capture intermediate adaptive processes that are not included in the current model. In addition, transcriptome profiles of Mtb from *in vivo* contexts (i.e., granuloma, caseum) remain a key limitation in discovering regulatory events of important microenvironments.

STAR★METHODS

Detailed methods are provided in the online version of this paper and include the following:

- KEY RESOURCES TABLE
- RESOURCE AVAILABILITY
  - Lead contact
  - Materials availability
  - Data and code availability
- EXPERIMENTAL MODEL AND STUDY PARTICIPANT DETAILS
- METHOD DETAILS
  - EGRIN 2.0 construction
  - Transposon mutant library sequencing
  - Permutation test for evaluating significance of overlap between corems and genes with reduced fitness
  - Gene expression profiling of CRISPRi-mediated mtrA knockdown
  - Gene expression profiling at netural or acidic pH
  - Gene expression profiling following antibiotic treatment
  - Processing and analysis of RNA-seq data
  - Differential expression analysis
  - Peptidoglycan labeling with fluorescent d-alanine analogues
  - Microscopy
  - Phenotyping CRISPRi-mediated mtrA knockdown
  - Antibiotic activity measurements
  - Time-kill assay
- QUANTIFICATION AND STATISTICAL ANALYSIS
- ADDITIONAL RESOURCES

SUPPLEMENTAL INFORMATION

Supplemental information can be found online at https://doi.org/10.1016/j.celrep.2023.112875.

ACKNOWLEDGMENTS

We gratefully acknowledge Sarah Fortune for kindly providing us with the PLJR962 and PLJR965 plasmids. We thank Michael VanNieuwenhze for kindly providing HADA and Malini Rajagopalan for providing pMZ3 and pMG129. We gratefully acknowledge Tanya Parish for synthesis of compounds 2 and 6. We thank members of the Baliga lab for critical discussions and feedback. This work is funded by the National Institute of Allergy and Infectious Diseases of the National Institutes of Health (R01AI128215, R01AI141953, and U19AI135976) and the Bill and Melinda Gates Foundation (INV-009322).

AUTHOR CONTRIBUTIONS

E.J.R.P. and N.S.B. conceived the study. A.N.B. and D.J.R. ran *cMonkey* and constructed the ensemble model. E.J.R.P. analyzed the model and all data represented. W.-J.W. provided assistance with ensemble analyses. R.M. analyzed Tn-seq data. A.K., J.D., V.S., W.C., M.P., and R.A.R. performed experiments. M.L.A.-O. helped with computational analyses. A.B. helped with designing experimental validations. E.J.R.P. and N.S.B. wrote the paper and supervised the study. All authors read the manuscript and approved its content.

DECLARATION OF INTERESTS

The authors declare no competing interest.

Received: August 16, 2021
Revised: April 21, 2023
Accepted: July 11, 2023
Published: August 4, 2023

REFERENCES

1. Zhu, J., Liu, Y.J., and Fortune, S.M. (2023). Spatiotemporal perspectives on tuberculosis chemotherapy. Curr. Opin. Microbiol. *72*, 102266. https://doi.org/10.1016/j.mib.2023.102266.
2. Warner, D.F., and Mizrahi, V. (2006). Tuberculosis chemotherapy: the influence of bacillary stress and damage response pathways on drug efficacy. Clin. Microbiol. Rev. *19*, 558–570. https://doi.org/10.1128/CMR.00060-05.
3. Brooks, A.N., Reiss, D.J., Allard, A., Wu, W.J., Salvanha, D.M., Plaisier, C.L., Chandrasekaran, S., Pan, M., Kaur, A., and Baliga, N.S. (2014). A system-level model for the microbial regulatory genome. Mol. Syst. Biol. *10*, 740. https://doi.org/10.15252/msb.20145160.
4. Bonneau, R., Facciotti, M.T., Reiss, D.J., Schmid, A.K., Pan, M., Kaur, A., Thorsson, V., Shannon, P., Johnson, M.H., Bare, J.C., et al. (2007). A predictive model for transcriptional control of physiology in a free living cell. Cell *131*, 1354–1365. https://doi.org/10.1016/j.cell.2007.10.053.
5. Reiss, D.J., Baliga, N.S., and Bonneau, R. (2006). Integrated biclustering of heterogeneous genome-wide datasets for the inference of global regulatory networks. BMC Bioinf. *7*, 280. https://doi.org/10.1186/1471-2105-7-280.
6. Reiss, D.J., Plaisier, C.L., Wu, W.J., and Baliga, N.S. (2015). cMonkey2: Automated, systematic, integrated detection of co-regulated gene modules for any organism. Nucleic Acids Res. *43*, e87. https://doi.org/10.1093/nar/gkv300.
7. Bonneau, R., Reiss, D.J., Shannon, P., Facciotti, M., Hood, L., Baliga, N.S., and Thorsson, V. (2006). The Inferelator: an algorithm for learning parsimonious regulatory networks from systems-biology data sets de novo. Genome Biol. *7*, R36. https://doi.org/10.1186/gb-2006-7-5-r36.
8. Plaisier, C.L., O'Brien, S., Bernard, B., Reynolds, S., Simon, Z., Toledo, C.M., Ding, Y., Reiss, D.J., Paddison, P.J., and Baliga, N.S. (2016). Causal Mechanistic Regulatory Network for Glioblastoma Deciphered Using Systems Genetics Network Analysis. Cell Syst. *3*, 172–186. https://doi.org/10.1016/j.cels.2016.06.006.
9. Peterson, E.J.R., Reiss, D.J., Turkarslan, S., Minch, K.J., Rustad, T., Plaisier, C.L., Longabaugh, W.J.R., Sherman, D.R., and Baliga, N.S. (2014). A high-resolution network model for global gene regulation in Mycobacterium tuberculosis. Nucleic Acids Res. *42*, 11291–11303. https://doi.org/10.1093/nar/gku777.
10. Turkarslan, S., Reiss, D.J., Gibbins, G., Su, W.L., Pan, M., Bare, J.C., Plaisier, C.L., and Baliga, N.S. (2011). Niche adaptation by expansion and reprogramming of general transcription factors. Mol. Syst. Biol. *7*, 554. https://doi.org/10.1038/msb.2011.87.
11. Ashworth, J., Coesel, S., Lee, A., Armbrust, E.V., Orellana, M.V., and Baliga, N.S. (2013). Genome-wide diel growth state transitions in the diatom

Thalassiosira pseudonana. Proc. Natl. Acad. Sci. USA *110*, 7518–7523. https://doi.org/10.1073/pnas.1300962110.

12. Rothchild, A.C., Sissons, J.R., Shafiani, S., Plaisier, C., Min, D., Mai, D., Gilchrist, M., Peschon, J., Larson, R.P., Bergthaler, A., et al. (2016). MiR-155-regulated molecular network orchestrates cell fate in the innate and adaptive immune response to Mycobacterium tuberculosis. Proc. Natl. Acad. Sci. USA *113*, E6172–E6181. https://doi.org/10.1073/pnas.1608255113.
13. López García de Lomana, A., Schäuble, S., Valenzuela, J., Imam, S., Carter, W., Bilgin, D.D., Yohn, C.B., Turkarslan, S., Reiss, D.J., Orellana, M.V., et al. (2015). Transcriptional program for nitrogen starvation-induced lipid accumulation in Chlamydomonas reinhardtii. Biotechnol. Biofuels *8*, 207. https://doi.org/10.1186/s13068-015-0391-z.
14. Neal, M.L., Wei, L., Peterson, E., Arrieta-Ortiz, M.L., Danziger, S.A., Baliga, N.S., Kaushansky, A., and Aitchison, J.D. (2021). A systems-level gene regulatory network model for Plasmodium falciparum. Nucleic Acids Res. *49*, 4891–4906. https://doi.org/10.1093/nar/gkaa1245.
15. Danziger, S.A., Ratushny, A.V., Smith, J.J., Saleem, R.A., Wan, Y., Arens, C.E., Armstrong, A.M., Sitko, K., Chen, W.M., Chiang, J.H., et al. (2014). Molecular mechanisms of system responses to novel stimuli are predictable from public data. Nucleic Acids Res. *42*, 1442–1460. https://doi.org/10.1093/nar/gkt938.
16. Schaible, U.E., Sturgill-Koszycki, S., Schlesinger, P.H., and Russell, D.G. (1998). Cytokine activation leads to acidification and increases maturation of Mycobacterium avium-containing phagosomes in murine macrophages. J. Immunol. *160*, 1290–1296.
17. Yates, R.M., Hermetter, A., and Russell, D.G. (2005). The kinetics of phagosome maturation as a function of phagosome/lysosome fusion and acquisition of hydrolytic activity. Traffic *6*, 413–420. https://doi.org/10.1111/j.1600-0854.2005.00284.x.
18. Kempker, R.R., Heinrichs, M.T., Nikolaishvili, K., Sabulua, I., Bablishvili, N., Gogishvili, S., Avaliani, Z., Tukvadze, N., Little, B., Bernheim, A., et al. (2017). Lung Tissue Concentrations of Pyrazinamide among Patients with Drug-Resistant Pulmonary Tuberculosis. Antimicrob. Agents Chemother. *61*, e00226-17. https://doi.org/10.1128/AAC.00226-17.
19. Lavin, R.C., and Tan, S. (2022). Spatial relationships of intra-lesion heterogeneity in Mycobacterium tuberculosis microenvironment, replication status, and drug efficacy. PLoS Pathog. *18*, e1010459. https://doi.org/10.1371/journal.ppat.1010459.
20. Baker, J.J., Dechow, S.J., and Abramovitch, R.B. (2019). Acid Fasting: Modulation of Mycobacterium tuberculosis Metabolism at Acidic pH. Trends Microbiol. *27*, 942–953. https://doi.org/10.1016/j.tim.2019.06.005.
21. Rohde, K.H., Abramovitch, R.B., and Russell, D.G. (2007). Mycobacterium tuberculosis invasion of macrophages: linking bacterial gene expression to environmental cues. Cell Host Microbe *2*, 352–364. https://doi.org/10.1016/j.chom.2007.09.006.
22. Tan, S., Sukumar, N., Abramovitch, R.B., Parish, T., and Russell, D.G. (2013). Mycobacterium tuberculosis responds to chloride and pH as synergistic cues to the immune status of its host cell. PLoS Pathog. *9*, e1003282. https://doi.org/10.1371/journal.ppat.1003282.
23. Abramovitch, R.B., Rohde, K.H., Hsu, F.F., and Russell, D.G. (2011). aprABC: a Mycobacterium tuberculosis complex-specific locus that modulates pH-driven adaptation to the macrophage phagosome. Mol. Microbiol. *80*, 678–694. https://doi.org/10.1111/j.1365-2958.2011.07601.x.
24. Zahrt, T.C., and Deretic, V. (2000). An essential two-component signal transduction system in Mycobacterium tuberculosis. J. Bacteriol. *182*, 3832–3838. https://doi.org/10.1128/jb.182.13.3832-3838.2000.
25. Via, L.E., Curcic, R., Mudd, M.H., Dhandayuthapani, S., Ulmer, R.J., and Deretic, V. (1996). Elements of signal transduction in Mycobacterium tuberculosis: in vitro phosphorylation and in vivo expression of the response regulator MtrA. J. Bacteriol. *178*, 3314–3321. https://doi.org/10.1128/jb.178.11.3314-3321.1996.
26. Curcic, R., Dhandayuthapani, S., and Deretic, V. (1994). Gene expression in mycobacteria: transcriptional fusions based on xylE and analysis of the promoter region of the response regulator mtrA from Mycobacterium tuberculosis. Mol. Microbiol. *13*, 1057–1064. https://doi.org/10.1111/j.1365-2958.1994.tb00496.x.
27. Dhandayuthapani, S., Via, L.E., Thomas, C.A., Horowitz, P.M., Deretic, D., and Deretic, V. (1995). Green fluorescent protein as a marker for gene expression and cell biology of mycobacterial interactions with macrophages. Mol. Microbiol. *17*, 901–912. https://doi.org/10.1111/j.1365-2958.1995.mmi_17050901.x.
28. Gorla, P., Plocinska, R., Sarva, K., Satsangi, A.T., Pandeeti, E., Donnelly, R., Dziadek, J., Rajagopalan, M., and Madiraju, M.V. (2018). MtrA Response Regulator Controls Cell Division and Cell Wall Metabolism and Affects Susceptibility of Mycobacteria to the First Line Antituberculosis Drugs. Front. Microbiol. *9*, 2839. https://doi.org/10.3389/fmicb.2018.02839.
29. Plocinska, R., Purushotham, G., Sarva, K., Vadrevu, I.S., Pandeeti, E.V.P., Arora, N., Plocinski, P., Madiraju, M.V., and Rajagopalan, M. (2012). Septal localization of the Mycobacterium tuberculosis MtrB sensor kinase promotes MtrA regulon expression. J. Biol. Chem. *287*, 23887–23899. https://doi.org/10.1074/jbc.M112.346544.
30. Fol, M., Chauhan, A., Nair, N.K., Maloney, E., Moomey, M., Jagannath, C., Madiraju, M.V.V.S., and Rajagopalan, M. (2006). Modulation of Mycobacterium tuberculosis proliferation by MtrA, an essential two-component response regulator. Mol. Microbiol. *60*, 643–657. https://doi.org/10.1111/j.1365-2958.2006.05137.x.
31. Rajagopalan, M., Dziedzic, R., Al Zayer, M., Stankowska, D., Ouimet, M.C., Bastedo, D.P., Marczynski, G.T., and Madiraju, M.V. (2010). Mycobacterium tuberculosis origin of replication and the promoter for immunodominant secreted antigen 85B are the targets of MtrA, the essential response regulator. J. Biol. Chem. *285*, 15816–15827. https://doi.org/10.1074/jbc.M109.040097.
32. Li, S., Poulton, N.C., Chang, J.S., Azadian, Z.A., DeJesus, M.A., Ruecker, N., Zimmerman, M.D., Eckartt, K.A., Bosch, B., Engelhart, C.A., et al. (2022). CRISPRi chemical genetics and comparative genomics identify genes mediating drug potency in Mycobacterium tuberculosis. Nat. Microbiol. *7*, 766–779. https://doi.org/10.1038/s41564-022-01130-y.
33. Minch, K.J., Rustad, T.R., Peterson, E.J.R., Winkler, J., Reiss, D.J., Ma, S., Hickey, M., Brabant, W., Morrison, B., Turkarslan, S., et al. (2015). The DNA-binding network of Mycobacterium tuberculosis. Nat. Commun. *6*, 5829. https://doi.org/10.1038/ncomms6829.
34. Kumar, A., Deshane, J.S., Crossman, D.K., Bolisetty, S., Yan, B.S., Kramnik, I., Agarwal, A., and Steyn, A.J.C. (2008). Heme oxygenase-1-derived carbon monoxide induces the Mycobacterium tuberculosis dormancy regulon. J. Biol. Chem. *283*, 18032–18039. https://doi.org/10.1074/jbc.M802274200.
35. Huang, D.W., Sherman, B.T., and Lempicki, R.A. (2009). Systematic and integrative analysis of large gene lists using DAVID bioinformatics resources. Nat. Protoc. *4*, 44–57. https://doi.org/10.1038/nprot.2008.211.
36. Kapopoulou, A., Lew, J.M., and Cole, S.T. (2011). The MycoBrowser portal: a comprehensive and manually annotated resource for mycobacterial genomes. Tuberculosis *91*, 8–13. https://doi.org/10.1016/j.tube.2010.09.006.
37. Paquette, S.M., Leinonen, K., and Longabaugh, W.J.R. (2016). BioTapestry now provides a web application and improved drawing and layout tools. F1000Research *5*, 39. https://doi.org/10.12688/f1000research.7620.1.
38. van Opijnen, T., and Camilli, A. (2013). Transposon insertion sequencing: a new tool for systems-level analysis of microorganisms. Nat. Rev. Microbiol. *11*, 435–442. https://doi.org/10.1038/nrmicro3033.
39. DeJesus, M.A., Ambadipudi, C., Baker, R., Sassetti, C., and Ioerger, T.R. (2015). TRANSIT–A Software Tool for Himar1 TnSeq Analysis. PLoS Comput. Biol. *11*, e1004401. https://doi.org/10.1371/journal.pcbi.1004401.

40. Via, L.E., Lin, P.L., Ray, S.M., Carrillo, J., Allen, S.S., Eum, S.Y., Taylor, K., Klein, E., Manjunatha, U., Gonzales, J., et al. (2008). Tuberculous granulomas are hypoxic in guinea pigs, rabbits, and nonhuman primates. Infect. Immun. *76*, 2333–2340. https://doi.org/10.1128/IAI.01515-07.

41. Voskuil, M.I., Schnappinger, D., Visconti, K.C., Harrell, M.I., Dolganov, G.M., Sherman, D.R., and Schoolnik, G.K. (2003). Inhibition of respiration by nitric oxide induces a Mycobacterium tuberculosis dormancy program. J. Exp. Med. *198*, 705–713. https://doi.org/10.1084/jem.20030205.

42. Voskuil, M.I., Bartek, I.L., Visconti, K., and Schoolnik, G.K. (2011). The response of mycobacterium tuberculosis to reactive oxygen and nitrogen species. Front. Microbiol. *2*, 105. https://doi.org/10.3389/fmicb.2011.00105.

43. Cortes, T., Schubert, O.T., Banaei-Esfahani, A., Collins, B.C., Aebersold, R., and Young, D.B. (2017). Delayed effects of transcriptional responses in Mycobacterium tuberculosis exposed to nitric oxide suggest other mechanisms involved in survival. Sci. Rep. *7*, 8208. https://doi.org/10.1038/s41598-017-08306-1.

44. Farhana, A., Guidry, L., Srivastava, A., Singh, A., Hondalus, M.K., and Steyn, A.J.C. (2010). Reductive stress in microbes: implications for understanding Mycobacterium tuberculosis disease and persistence. Adv. Microb. Physiol. *57*, 43–117. https://doi.org/10.1016/B978-0-12-381045-8.00002-3.

45. Bhat, S.A., Iqbal, I.K., and Kumar, A. (2016). Imaging the NADH:NAD(+) Homeostasis for Understanding the Metabolic Response of Mycobacterium to Physiologically Relevant Stresses. Front. Cell. Infect. Microbiol. *6*, 145. https://doi.org/10.3389/fcimb.2016.00145.

46. Botella, H., Vaubourgeix, J., Lee, M.H., Song, N., Xu, W., Makinoshima, H., Glickman, M.S., and Ehrt, S. (2017). Mycobacterium tuberculosis protease MarP activates a peptidoglycan hydrolase during acid stress. EMBO J. *36*, 536–548. https://doi.org/10.15252/embj.201695028.

47. Mohn, W.W., van der Geize, R., Stewart, G.R., Okamoto, S., Liu, J., Dijkhuizen, L., and Eltis, L.D. (2008). The actinobacterial mce4 locus encodes a steroid transporter. J. Biol. Chem. *283*, 35368–35374. https://doi.org/10.1074/jbc.M805496200.

48. Park, H.D., Guinn, K.M., Harrell, M.I., Liao, R., Voskuil, M.I., Tompa, M., Schoolnik, G.K., and Sherman, D.R. (2003). Rv3133c/dosR is a transcription factor that mediates the hypoxic response of Mycobacterium tuberculosis. Mol. Microbiol. *48*, 833–843.

49. Parish, T., Smith, D.A., Kendall, S., Casali, N., Bancroft, G.J., and Stoker, N.G. (2003). Deletion of two-component regulatory systems increases the virulence of Mycobacterium tuberculosis. Infect. Immun. *71*, 1134–1140. https://doi.org/10.1128/iai.71.3.1134-1140.2003.

50. Robertson, D., Carroll, P., and Parish, T. (2007). Rapid recombination screening to test gene essentiality demonstrates that pyrH is essential in Mycobacterium tuberculosis. Tuberculosis *87*, 450–458. https://doi.org/10.1016/j.tube.2007.05.015.

51. Rock, J.M., Hopkins, F.F., Chavez, A., Diallo, M., Chase, M.R., Gerrick, E.R., Pritchard, J.R., Church, G.M., Rubin, E.J., Sassetti, C.M., et al. (2017). Programmable transcriptional repression in mycobacteria using an orthogonal CRISPR interference platform. Nat. Microbiol. *2*, 16274. https://doi.org/10.1038/nmicrobiol.2016.274.

52. Alland, D., Steyn, A.J., Weisbrod, T., Aldrich, K., and Jacobs, W.R., Jr. (2000). Characterization of the Mycobacterium tuberculosis iniBAC promoter, a promoter that responds to cell wall biosynthesis inhibition. J. Bacteriol. *182*, 1802–1811. https://doi.org/10.1128/jb.182.7.1802-1811.2000.

53. Grant, C.E., Bailey, T.L., and Noble, W.S. (2011). FIMO: scanning for occurrences of a given motif. Bioinformatics *27*, 1017–1018. https://doi.org/10.1093/bioinformatics/btr064.

54. Gupta, S., Stamatoyannopoulos, J.A., Bailey, T.L., and Noble, W.S. (2007). Quantifying similarity between motifs. Genome Biol. *8*, R24. https://doi.org/10.1186/gb-2007-8-2-r24.

55. Dragset, M.S., Ioerger, T.R., Zhang, Y.J., Mærk, M., Ginbot, Z., Sacchettini, J.C., Flo, T.H., Rubin, E.J., and Steigedal, M. (2019). Genome-wide Phenotypic Profiling Identifies and Categorizes Genes Required for Mycobacterial Low Iron Fitness. Sci. Rep. *9*, 11394. https://doi.org/10.1038/s41598-019-47905-y.

56. Kang, C.M., Nyayapathy, S., Lee, J.Y., Suh, J.W., and Husson, R.N. (2008). Wag31, a homologue of the cell division protein DivIVA, regulates growth, morphology and polar cell wall synthesis in mycobacteria. Microbiology (Read.) *154*, 725–735. https://doi.org/10.1099/mic.0.2007/014076-0.

57. Wu, K.J., Zhang, J., Baranowski, C., Leung, V., Rego, E.H., Morita, Y.S., Rubin, E.J., and Boutte, C.C. (2018). Characterization of Conserved and Novel Septal Factors in Mycobacterium smegmatis. J. Bacteriol. *200*, e00649-17. https://doi.org/10.1128/JB.00649-17.

58. Hett, E.C., Chao, M.C., Deng, L.L., and Rubin, E.J. (2008). A mycobacterial enzyme essential for cell division synergizes with resuscitation-promoting factor. PLoS Pathog. *4*, e1000001. https://doi.org/10.1371/journal.ppat.1000001.

59. Hett, E.C., Chao, M.C., Steyn, A.J., Fortune, S.M., Deng, L.L., and Rubin, E.J. (2007). A partner for the resuscitation-promoting factors of Mycobacterium tuberculosis. Mol. Microbiol. *66*, 658–668. https://doi.org/10.1111/j.1365-2958.2007.05945.x.

60. Hett, E.C., Chao, M.C., and Rubin, E.J. (2010). Interaction and modulation of two antagonistic cell wall enzymes of mycobacteria. PLoS Pathog. *6*, e1001020. https://doi.org/10.1371/journal.ppat.1001020.

61. Chao, M.C., Kieser, K.J., Minami, S., Mavrici, D., Aldridge, B.B., Fortune, S.M., Alber, T., and Rubin, E.J. (2013). Protein complexes and proteolytic activation of the cell wall hydrolase RipA regulate septal resolution in mycobacteria. PLoS Pathog. *9*, e1003197. https://doi.org/10.1371/journal.ppat.1003197.

62. Kieser, K.J., Baranowski, C., Chao, M.C., Long, J.E., Sassetti, C.M., Waldor, M.K., Sacchettini, J.C., Ioerger, T.R., and Rubin, E.J. (2015). Peptidoglycan synthesis in Mycobacterium tuberculosis is organized into networks with varying drug susceptibility. Proc. Natl. Acad. Sci. USA *112*, 13087–13092. https://doi.org/10.1073/pnas.1514135112.

63. Al Zayer, M., Stankowska, D., Dziedzic, R., Sarva, K., Madiraju, M.V., and Rajagopalan, M. (2011). Mycobacterium tuberculosis mtrA merodiploid strains with point mutations in the signal-receiving domain of MtrA exhibit growth defects in nutrient broth. Plasmid *65*, 210–218. https://doi.org/10.1016/j.plasmid.2011.01.002.

64. Vollmer, W. (2012). Bacterial growth does require peptidoglycan hydrolases. Mol. Microbiol. *86*, 1031–1035. https://doi.org/10.1111/mmi.12059.

65. Do, T., Page, J.E., and Walker, S. (2020). Uncovering the activities, biological roles, and regulation of bacterial cell wall hydrolases and tailoring enzymes. J. Biol. Chem. *295*, 3347–3361. https://doi.org/10.1074/jbc.REV119.010155.

66. Vollmer, W., Joris, B., Charlier, P., and Foster, S. (2008). Bacterial peptidoglycan (murein) hydrolases. FEMS Microbiol. Rev. *32*, 259–286. https://doi.org/10.1111/j.1574-6976.2007.00099.x.

67. Machowski, E.E., Senzani, S., Ealand, C., and Kana, B.D. (2014). Comparative genomics for mycobacterial peptidoglycan remodelling enzymes reveals extensive genetic multiplicity. BMC Microbiol. *14*, 75. https://doi.org/10.1186/1471-2180-14-75.

68. Böth, D., Schneider, G., and Schnell, R. (2011). Peptidoglycan remodeling in Mycobacterium tuberculosis: comparison of structures and catalytic activities of RipA and RipB. J. Mol. Biol. *413*, 247–260. https://doi.org/10.1016/j.jmb.2011.08.014.

69. Böth, D., Steiner, E.M., Izumi, A., Schneider, G., and Schnell, R. (2014). RipD (Rv1566c) from Mycobacterium tuberculosis: adaptation of an NlpC/p60 domain to a non-catalytic peptidoglycan-binding function. Biochem. J. *457*, 33–41. https://doi.org/10.1042/BJ20131227.

70. Bellinzoni, M., Haouz, A., Miras, I., Magnet, S., André-Leroux, G., Mukherjee, R., Shepard, W., Cole, S.T., and Alzari, P.M. (2014). Structural studies suggest a peptidoglycan hydrolase function for the Mycobacterium tuberculosis Tat-secreted protein Rv2525c. J. Struct. Biol. *188*, 156–164. https://doi.org/10.1016/j.jsb.2014.09.003.

71. Kuru, E., Tekkam, S., Hall, E., Brun, Y.V., and Van Nieuwenhze, M.S. (2015). Synthesis of fluorescent D-amino acids and their use for probing peptidoglycan synthesis and bacterial growth in situ. Nat. Protoc. *10*, 33–52. https://doi.org/10.1038/nprot.2014.197.

72. Aldridge, B.B., Fernandez-Suarez, M., Heller, D., Ambravaneswaran, V., Irimia, D., Toner, M., and Fortune, S.M. (2012). Asymmetry and aging of mycobacterial cells lead to variable growth and antibiotic susceptibility. Science *335*, 100–104. https://doi.org/10.1126/science.1216166.

73. Meniche, X., Otten, R., Siegrist, M.S., Baer, C.E., Murphy, K.C., Bertozzi, C.R., and Sassetti, C.M. (2014). Subpolar addition of new cell wall is directed by DivIVA in mycobacteria. Proc. Natl. Acad. Sci. USA *111*, E3243–E3251. https://doi.org/10.1073/pnas.1402158111.

74. Banerjee, S.K., Kumar, M., Alokam, R., Sharma, A.K., Chatterjee, A., Kumar, R., Sahu, S.K., Jana, K., Singh, R., Yogeeswari, P., et al. (2016). Targeting multiple response regulators of Mycobacterium tuberculosis augments the host immune response to infection. Sci. Rep. *6*, 25851. https://doi.org/10.1038/srep25851.

75. Johnson, E.O., LaVerriere, E., Office, E., Stanley, M., Meyer, E., Kawate, T., Gomez, J.E., Audette, R.E., Bandyopadhyay, N., Betancourt, N., et al. (2019). Large-scale chemical-genetics yields new M. tuberculosis inhibitor classes. Nature *571*, 72–78. https://doi.org/10.1038/s41586-019-1315-z.

76. Evans, J.C., Trujillo, C., Wang, Z., Eoh, H., Ehrt, S., Schnappinger, D., Boshoff, H.I.M., Rhee, K.Y., Barry, C.E., 3rd, and Mizrahi, V. (2016). Validation of CoaBC as a Bactericidal Target in the Coenzyme A Pathway of Mycobacterium tuberculosis. ACS Infect. Dis. *2*, 958–968. https://doi.org/10.1021/acsinfecdis.6b00150.

77. Abrahams, G.L., Kumar, A., Savvi, S., Hung, A.W., Wen, S., Abell, C., Barry, C.E., 3rd, Sherman, D.R., Boshoff, H.I.M., and Mizrahi, V. (2012). Pathway-selective sensitization of Mycobacterium tuberculosis for target-based whole-cell screening. Chem. Biol. *19*, 844–854. https://doi.org/10.1016/j.chembiol.2012.05.020.

78. Dar, D., Dar, N., Cai, L., and Newman, D.K. (2021). Spatial transcriptomics of planktonic and sessile bacterial populations at single-cell resolution. Science *373*, eabi4882. https://doi.org/10.1126/science.abi4882.

79. Tagkopoulos, I., Liu, Y.C., and Tavazoie, S. (2008). Predictive behavior within microbial genetic networks. Science *320*, 1313–1317.

80. Baliga, N.S. (2008). Systems biology: the scale of prediction. Science *320*, 1297–1298.

81. Healy, C., Gouzy, A., and Ehrt, S. (2020). Peptidoglycan Hydrolases RipA and Ami1 Are Critical for Replication and Persistence of Mycobacterium tuberculosis in the Host. mBio *11*, e03315-19. https://doi.org/10.1128/mBio.03315-19.

82. Lee, T.K., and Huang, K.C. (2013). The role of hydrolases in bacterial cell-wall growth. Curr. Opin. Microbiol. *16*, 760–766. https://doi.org/10.1016/j.mib.2013.08.005.

83. Boutte, C.C., Baer, C.E., Papavinasasundaram, K., Liu, W., Chase, M.R., Meniche, X., Fortune, S.M., Sassetti, C.M., Ioerger, T.R., and Rubin, E.J. (2016). A cytoplasmic peptidoglycan amidase homologue controls mycobacterial cell wall synthesis. Elife *5*, e14590. https://doi.org/10.7554/eLife.14590.

84. Chatterjee, A., Sharma, A.K., Mahatha, A.C., Banerjee, S.K., Kumar, M., Saha, S., Basu, J., and Kundu, M. (2018). Global mapping of MtrA-binding sites links MtrA to regulation of its targets in Mycobacterium tuberculosis. Microbiology (Read.) *164*, 99–110. https://doi.org/10.1099/mic.0.000585.

85. Peterson, E.J.R., Abidi, A.A., Arrieta-Ortiz, M.L., Aguilar, B., Yurkovich, J.T., Kaur, A., Pan, M., Srinivas, V., Shmulevich, I., and Baliga, N.S. (2020). Intricate Genetic Programs Controlling Dormancy in Mycobacterium tuberculosis. Cell Rep. *31*, 107577. https://doi.org/10.1016/j.celrep.2020.107577.

86. Hoch, J.A. (2000). Two-component and phosphorelay signal transduction. Curr. Opin. Microbiol. *3*, 165–170. https://doi.org/10.1016/s1369-5274(00)00070-9.

87. Stock, A.M., Robinson, V.L., and Goudreau, P.N. (2000). Two-component signal transduction. Annu. Rev. Biochem. *69*, 183–215. https://doi.org/10.1146/annurev.biochem.69.1.183.

88. Malhotra, V., Okon, B.P., Satsangi, A.T., Das, S., Waturuocha, U.W., Vashist, A., Clark-Curtiss, J.E., and Saini, D.K. (2022). Mycobacterium tuberculosis PknK Substrate Profiling Reveals Essential Transcription Terminator Protein Rho and Two-Component Response Regulators PrrA and MtrA as Novel Targets for Phosphorylation. Microbiol. Spectr. *10*, e0135421. https://doi.org/10.1128/spectrum.01354-21.

89. Carette, X., Platig, J., Young, D.C., Helmel, M., Young, A.T., Wang, Z., Potluri, L.P., Moody, C.S., Zeng, J., Prisic, S., et al. (2018). Multisystem Analysis of Mycobacterium tuberculosis Reveals Kinase-Dependent Remodeling of the Pathogen-Environment Interface. mBio *9*, e02333-17. https://doi.org/10.1128/mBio.02333-17.

90. Horstmann, N., Saldaña, M., Sahasrabhojane, P., Yao, H., Su, X., Thompson, E., Koller, A., and Shelburne, S.A., 3rd. (2014). Dual-site phosphorylation of the control of virulence regulator impacts group a streptococcal global gene expression and pathogenesis. PLoS Pathog. *10*, e1004088. https://doi.org/10.1371/journal.ppat.1004088.

91. Botella, H., Yang, G., Ouerfelli, O., Ehrt, S., Nathan, C.F., and Vaubourgeix, J. (2017). Distinct Spatiotemporal Dynamics of Peptidoglycan Synthesis between Mycobacterium smegmatis and Mycobacterium tuberculosis. mBio *8*, e01183-17. https://doi.org/10.1128/mBio.01183-17.

92. Senzani, S., Li, D., Bhaskar, A., Ealand, C., Chang, J., Rimal, B., Liu, C., Joon Kim, S., Dhar, N., and Kana, B. (2017). An Amidase_3 domain-containing N-acetylmuramyl-L-alanine amidase is required for mycobacterial cell division. Sci. Rep. *7*, 1140. https://doi.org/10.1038/s41598-017-01184-7.

93. Patru, M.M., and Pavelka, M.S., Jr. (2010). A role for the class A penicillin-binding protein PonA2 in the survival of Mycobacterium smegmatis under conditions of nonreplication. J. Bacteriol. *192*, 3043–3054. https://doi.org/10.1128/JB.00025-10.

94. Brooks, A.N., Turkarslan, S., Beer, K.D., Lo, F.Y., and Baliga, N.S. (2011). Adaptation of cells to new environments. Wiley Interdiscip Rev Syst Biol Med *3*, 544–561. https://doi.org/10.1002/wsbm.136.

95. López-Maury, L., Marguerat, S., and Bähler, J. (2008). Tuning gene expression to changing environments: from rapid responses to evolutionary adaptation. Nat. Rev. Genet. *9*, 583–593. https://doi.org/10.1038/nrg2398.

96. Stumpf, C.R., Moreno, M.V., Olshen, A.B., Taylor, B.S., and Ruggero, D. (2013). The translational landscape of the mammalian cell cycle. Mol. Cell *52*, 574–582. https://doi.org/10.1016/j.molcel.2013.09.018.

97. Morris, R.P., Nguyen, L., Gatfield, J., Visconti, K., Nguyen, K., Schnappinger, D., Ehrt, S., Liu, Y., Heifets, L., Pieters, J., et al. (2005). Ancestral antibiotic resistance in Mycobacterium tuberculosis. Proc. Natl. Acad. Sci. USA *102*, 12200–12205. https://doi.org/10.1073/pnas.0505446102.

98. Schrader, S.M., Botella, H., Jansen, R., Ehrt, S., Rhee, K., Nathan, C., and Vaubourgeix, J. (2021). Multiform antimicrobial resistance from a metabolic mutation. Sci. Adv. *7*, eabh2037. https://doi.org/10.1126/sciadv.abh2037.

99. Vignali, M., Armour, C.D., Chen, J., Morrison, R., Castle, J.C., Biery, M.C., Bouzek, H., Moon, W., Babak, T., Fried, M., et al. (2011). NSR-seq transcriptional profiling enables identification of a gene signature of Plasmodium falciparum parasites infecting children. J. Clin. Invest. *121*, 1119–1129. https://doi.org/10.1172/JCI43457.

100. Schneider, C.A., Rasband, W.S., and Eliceiri, K.W. (2012). NIH Image to ImageJ: 25 years of image analysis. Nat. Methods *9*, 671–675. https://doi.org/10.1038/nmeth.2089.

101. Dehal, P.S., Joachimiak, M.P., Price, M.N., Bates, J.T., Baumohl, J.K., Chivian, D., Friedland, G.D., Huang, K.H., Keller, K., Novichkov, P.S., et al. (2010). MicrobesOnline: an integrated portal for comparative and functional genomics. Nucleic Acids Res. *38*, D396–D400. https://doi.org/10.1093/nar/gkp919.

102. Sanz, J., Navarro, J., Arbués, A., Martín, C., Marijuán, P.C., and Moreno, Y. (2011). The transcriptional regulatory network of Mycobacterium tuberculosis. PLoS One *6*, e22178. https://doi.org/10.1371/journal.pone.0022178.

103. Long, J.E., DeJesus, M., Ward, D., Baker, R.E., Ioerger, T., and Sassetti, C.M. (2015). Identifying essential genes in Mycobacterium tuberculosis by global phenotypic profiling. Methods Mol. Biol. *1279*, 79–95. https://doi.org/10.1007/978-1-4939-2398-4_6.

104. van Opijnen, T., Bodi, K.L., and Camilli, A. (2009). Tn-seq: high-throughput parallel sequencing for fitness and genetic interaction studies in microorganisms. Nat. Methods *6*, 767–772. https://doi.org/10.1038/nmeth.1377.

105. Langmead, B., and Salzberg, S.L. (2012). Fast gapped-read alignment with Bowtie 2. Nat. Methods *9*, 357–359. https://doi.org/10.1038/nmeth.1923.

106. Breitling, R., Armengaud, P., Amtmann, A., and Herzyk, P. (2004). Rank products: a simple, yet powerful, new method to detect differentially regulated genes in replicated microarray experiments. FEBS Lett. *573*, 83–92. https://doi.org/10.1016/j.febslet.2004.07.055.

107. Tusher, V.G., Tibshirani, R., and Chu, G. (2001). Significance analysis of microarrays applied to the ionizing radiation response. Proc. Natl. Acad. Sci. USA *98*, 5116–5121. https://doi.org/10.1073/pnas.091062498.

108. Robinson, M.D., and Smyth, G.K. (2008). Small-sample estimation of negative binomial dispersion, with applications to SAGE data. Biostatistics *9*, 321–332. https://doi.org/10.1093/biostatistics/kxm030.

109. Love, M.I., Huber, W., and Anders, S. (2014). Moderated estimation of fold change and dispersion for RNA-seq data with DESeq2. Genome Biol. *15*, 550. https://doi.org/10.1186/s13059-014-0550-8.

110. Virtanen, P., Gommers, R., Oliphant, T.E., Haberland, M., Reddy, T., Cournapeau, D., Burovski, E., Peterson, P., Weckesser, W., Bright, J., et al. (2020). SciPy 1.0: fundamental algorithms for scientific computing in Python. Nat. Methods *17*, 261–272. https://doi.org/10.1038/s41592-019-0686-2.

STAR★METHODS

KEY RESOURCES TABLE

REAGENT or RESOURCE	SOURCE	IDENTIFIER
**Bacterial and virus strains**		
H37Rv (*M. tuberculosis*)	D. Sherman lab	N/A
mc2155 (*M. smegmatis*)	A. Bhatt lab	N/A
**Biological samples**		
φMycoMarT7 phagemid	D. Sherman lab	N/A
**Chemicals, peptides, and recombinant proteins**		
HADA (HCC-amino-d-alanine)	M. VanNieuwenhze lab	N/A
pMZ3: plasmid expressing $MtrA_{D13A}$	M. Rajagopalan lab	N/A
pMG129: plasmid expressing $MtrA_{D56N}$	M. Rajagopalan lab	N/A
Compound 2	T. Parish lab	
Compound 6	T. Parish lab	
**Critical commercial assays**		
NextSeq 500/550 High output kit	Illumina	FC-404-2002
RiboZero kit bacteria	Illumina	MRZB12424
TruSeq stranded mRNA library prep kit	Illumina	RS-122-2103
**Deposited data**		
Raw and analyzed data	This paper	NCBI GEO: GSE166806
Raw Tn-seq data	This paper	SRA Bioproject: PRJNA701946
EGRIN 2.0 model of Mtb	This paper	http://networks.systemsbiology.net/mtb/
**Experimental models: Organisms/strains**		
Transposon library in *M. tuberculosis* using φMycoMarT7 phagemid	This study	N/A
M. tuberculosis: pMZ3 (expressing $MtrA_{D13A}$)	Al Zayer et al.[63]	N/A
M. tuberculosis: pMG129 (expressing $MtrA_{D56N}$)	Fol et al.[30]	N/A
M. tuberculosis: CRISRPi knockdown of MtrA	This study	N/A
M. smegmatis: CRISPRi knockdown of MtrA	This study	N/A
**Oligonucleotides**		
CRISPRi sgRNAs, see Table S1	This study	N/A
**Recombinant DNA**		
pMZ3: plasmid expressing $MtrA_{D13A}$	M. Rajagopalan lab	N/A
pMG129: plasmid expressing $MtrA_{D56N}$	M. Rajagopalan lab	N/A
PLJR962: plasmid for CRISPRi in *M. tuberculosis*	S. Fortune lab	N/A
PLJR965: plasmid for CRISPRI in *M. smegmatis*	S. Fortune lab	N/A
**Software and algorithms**		
DuffyNGS	Vignali et al.[99]	http://networks.systemsbiology.net/mtb/
cMonkey2	Reiss et al.[6]	https://github.com/baliga-lab/cmonkey2
EGRIN2 API	Brooks et al.[3]	https://github.com/baliga-lab/egrin2api_mtu
EGRIN2 tools	Brooks et al.[3]	https://github.com/baliga-lab/egrin2-tools
TRANSIT	DeJesus et al.[39]	https://github.com/mad-lab/transit
Tn-seq processing	This study	https://doi.org/10.5281/zenodo.8088502 https://github.com/baliga-lab/tnseq_processing
GraphPad Prism, Version 9.5.1	GraphPad Software	https://www.graphpad.com/
ImageJ	Schneider et al.[100]	https://imagej.nih.gov/ij/

RESOURCE AVAILABILITY

Lead contact

Further information and requests for resources and reagents should be directed to and will be fulfilled by the lead contact, Nitin Baliga (nitin.baliga@isbscience.org).

Materials availability

This study did not generate new unique reagents.

Data and code availability

- RNA-seq data generated in this study has been deposited at NCBI GEO (NCBI GEO: GSE166806), and raw Tn-seq data generated in this study has been deposited at SRA (SRA Bioproject: PRJNA701946) Accession numbers are also listed in the key resources table.
- All original code has been deposited at Github (https://doi.org/10.5281/zenodo.8088502) and is publicly available as of the date of publication. The DOI is listed in the key resources table.
- Any additional information required to reanalyze the data reported in this paper is available from the lead contact upon request.

EXPERIMENTAL MODEL AND STUDY PARTICIPANT DETAILS

All *M. tuberculosis* strains are derivatives of H37Rv; all *M. smegmatis* strains are derivatives of mc^2155. *M. tuberculosis* and *M. smegmatis* were grown at 37°C in Middlebrook 7H9 broth or 7H10 plates supplemented with 0.2% glycerol, 0.05% Tween-80, and 10% ADC (liquid media) or OADC (plates), with aeration. Where indicated, anhydrotetracycline (ATc) was used at 100 ng/ml. For comparison of neutral and acidic pH media, the neutral media was 7H9 broth supplemented with 0.2% glycerol, 0.05% tyloxapol, and ADC buffered with 100 mM 3-(N-morpholino) propanesulfonic acid (MOPS) to pH 7. The acidic pH media was the same 7H9-rich media buffered with 100 mM 2-(N-morpholino) ethanesulfonic acid (MES) to pH 5.6. Frozen cells were inoculated into standard 7H9-rich media, grown to mid-log phase (OD_{600} ~0.5-0.7), washed in 1x PBS three times, and diluted into either neutral or acidic pH media at a starting density of OD_{600} = 0.05.

METHOD DETAILS

EGRIN 2.0 construction

EGRIN 2.0 was constructed as an ensemble of many individual EGRIN models (~300 for *M. tuberculosis*). Each EGRIN model was constructed using *cMonkey2*.[6] The input to *cMonkey2* was 1,861 transcriptome profiles with metadata collected about each experiment to annotate the environmental context (termed condition block, Data S1). Other input to *cMonkey2* were upstream regions of all genes, and functional association networks, including operon predictions from MicrobesOnline[101] and functional protein interactions from EMBL String databases.[102] We integrated the EGRIN models and mined the ensemble to discover frequently reoccurring features and associations. We refer to the modules detected by our procedure as co-regulated modules, or corems, the frequently re-occurring *de novo* cis-regulatory motifs as gene regulatory elements, or GREs.[3] Full description of the algorithms and each post-processing step is documented in Supplementary Information of Brooks et al[3].

Transposon mutant library sequencing

Six transposon mutant libraries were constructed using the φMycoMarT7 phagemid in *M. tuberculosis* H37Rv. For T0 samples, the mutant libraries were grown to mid-log phase in 7H9-rich media, then diluted to OD_{600} = 0.1 in PBS with 0.05% Tween-80 and plated onto 245 mm x 245 mm 7H10 plates supplemented with kanamycin (50 μg/ml) with ~30-40 thousand bacteria per plate. Additional samples were were grown to mid-log phase in 7H9-rich media, then diluted to OD_{600} = 0.1 in 7H9-rich media at neutral (pH 7.0) or acidic pH (pH 5.6) media for 72 hours and then plated. libraries In another experiment, the mutant libraries were diluted back in 7H9-rich media with or without 0.05% SDS. After 3 weeks of growth, colonies were scraped, and bacteria were resuspended in buffer for lysis and genomic DNA isolation. The mutant composition was determined by sequencing amplicons of the transposon-genome junctions following the protocol outlined by Long et al[103]. Paired-end reads were run on an Illumina HiSeq 2500 at the Genomics Services Core at Fred Hutchinson Cancer Research Center. Mapping and quantification of transposon insertions sites was done using *TRANSIT* analysis platform.[39] The change in fitness 'delta fitness' between conditions (*i.e.*, acidic pH vs neutral pH) for all genes was determined following the strategy of van Opijnen et al.[104] using a custom processing pipeline, full description and code is available at https://github.com/baliga-lab/tnseq_processing and http://networks.systemsbiology.net/mtb/software. The raw Tn-seq fastq sequence data files are deposited in the Sequence Read Archive database under accession SRA Bioproject: PRJNA701946.

Permutation test for evaluating significance of overlap between corems and genes with reduced fitness

The genes with reduced delta fitness in stress (either acidic pH or SDS treatment) were permutated 1000 times to generate shuffled gene clusters. In each permutation, the produced shuffled gene clusters had the same size as corem 71. Then, the average delta fitness for each shuffled gene set was computed and compared to the average delta fitness for corem 71. The overall permutation test *P* was computed as the proportion of cases (out of 1000 permutations) in which the average delta fitness was equal or lower than the observed value in corem 71.

Gene expression profiling of CRISPRi-mediated mtrA knockdown

In biological quadruplicate, cultures were grown to mid-log phase in 7H9-rich media with Kanamycin (50 μg/ml) and then diluted back in the presence or absence of 100 ng/mL ATc. Knockdown was allowed to proceed for 14 hours (*M. smegmatis*) or 4 days (*M. tuberculosis*), at which time samples were collected by centrifugation at high speed for 5 min, discarding supernatant and immediately flash freezing the cell pellet in liquid nitrogen. Cell pellets were stored at -80°C until RNA extraction was performed as previously described.[44]

Gene expression profiling at netural or acidic pH

To investigate gene expression changes of *M. tuberculosis* at acidic pH, cultures of *M. tuberculosis* (wildtype and merodiploid strains containing pMZ3 expressing $mtrA_{D13A}$ or pMG129 expressing $mtrA_{D56N}$) were cultured in standard 7H9-rich media to mid-log phase, washed three times in 1x PBS and diluted back to OD_{600} = 0.1 into either neutral or acidic pH media described above. Samples, in biological triplicate, were collected 4, 8, and 24 h after transfer to neutral or acidic pH media. Samples were centrifuged at high speed for 5 min, supernatant was discarded and the cell pellet was immediately flash frozen in liquid nitrogen. Cell pellets were stored at -80°C until RNA extraction was performed as previously described.[44]

Gene expression profiling following antibiotic treatment

Wildtype *M. tuberculosis* was cultured in standard 7H9-rich media, then diluted back to OD_{600} = 0.1 in 7H9-rich media containing either DMSO control, 34 μg/ml Compound 2 or 80 μg/ml Compound 6. Samples, in biological triplicate, were collected after 72 h. Samples were centrifuged at high speed for 5 min, supernatant was discarded and the cell pellet was immediately flash frozen in liquid nitrogen. Cell pellets were stored at -80°C until RNA extraction was performed as previously described.[44]

Processing and analysis of RNA-seq data

Total RNA samples were depleted of ribosomal RNA using the Ribo-Zero Bacteria rRNA Removal Kit (Illumina, San Diego, CA). Quality and purity of mRNA samples was determined with 2100 Bioanalyzer (Agilent, Santa Clara, CA). Samples were prepared with TrueSeq Stranded mRNA HT library preparation kit (Illumina, San Diego, CA). All samples were sequenced on the NextSeq sequencing instrument in a high output 150 v2 flow cell. Paired-end 75 bp reads were checked for technical artifacts using Illumina default quality filtering steps. Raw FASTQ read data were processed using the R package DuffyNGS.[99] Briefly, raw reads were passed through a 2-stage alignment pipeline: (i) a pre-alignment stage to filter out unwanted transcripts, such as rRNA; and (ii) a main genomic alignment stage against the genome of interest. Reads were aligned to *M. tuberculosis H37Rv* (ASM19595v2) or *M. smegmatis mc^{2}155* (ASM1500v1) with Bowtie 2,[105] using the command line option "very-sensitive." BAM files from stage (ii) were converted into read depth wiggle tracks that recorded both uniquely mapped and multiply mapped reads to each of the forward and reverse strands of the genome(s) at single-nucleotide resolution. Gene transcript abundance was then measured by summing total reads landing inside annotated gene boundaries, expressed as both RPKM and raw read counts. All RNA-seq data (raw and processed data) generated for this study are publicly available at the Gene Expression Omnibus under accession numbers NCBI GEO: GSE166806.

Differential expression analysis

We used a panel of 5 DE tools compiled in DuffyNGS to identify gene expression changes as previously described. The tools included (i) RoundRobin (in-house); (ii) RankProduct[106]; (iii) significance analysis of microarrays (SAM)[107]; (iv) EdgeR[108]; and (v) DESeq2.[109] Each DE tool was called with appropriate default parameters and operated on the same set of transcription results, using RPKM abundance units for RoundRobin, RankProduct, and SAM and raw read count abundance units for DESeq2 and EdgeR. Each DE tool's explicit measurements of differential expression (fold-change) and significance (*P*) were similarly combined via appropriate averaging (arithmetic and geometric mean, respectively).

Peptidoglycan labeling with fluorescent d-alanine analogues

HADA (HCC-amino-d-alanine) was synthesized by Michael VanNieuwenhze at Indiana University using methods previously published.[71] Cultures were grown to mid-log phase in 7H9-rich media with Kanamycin (50 μg/ml) and then diluted back in the presence or absence of ATc to induce CRISPRi-mediated *mtrA* knockdown. Knockdown was allowed to proceed for 14 hours (*M. smegmatis*) or 4 days (*M. tuberculosis*). Cultures were then inoculated at an OD600 of 0.1 to 0.3 into 7H9 medium supplemented with 1 mM HADA

and incubated for 3.5 hours (*M. smegmatis*) or 20 hours (*M. tuberculosis*). Bacterial suspension were washed 3 times with PBS-0.05% Tween-80 and fixed with paraformaldehyde for 30 min (*M. smegmatis*) or 4 hours (*M. tuberculosis*), to ensure bacterial death for further imaging outside a contained environment.

Microscopy

Fixed bacterial suspensions were mixed with the same volume of mounting medium and 10 μl amounts were spread on microscope slides and covered with cover glasses. Microscopy imaging was performed using SP8 Lightning super-resolution microscope (Leica Microsystems). Images were analyzed using ImageJ software.[100]

Phenotyping CRISPRi-mediated mtrA knockdown

To visually monitor the effects of CRIPSRi-mediated mtrA knockdown, cultures were grown to mid-log phase in 7H9-rich media with Kanamycin (50 μg/ml) and spotted on solid media with or without ATc. Plates were incubated at 37°C for 5 days (*M. smegmatis*) or 21 days (*M. tuberculosis*). Knockdown of *mtrA* was also quantified by RT-qPCR. CRISPRi strains were in the presence or absence of ATc (100 ng/ml) overnight (*M. smegmatis*) or for 5 days (*M. tuberculosis*) before samples were collected for total RNA extraction was performed as previously described.[44] Residual genomic DNA was removed by DNase treatment and 3 μg RNA per sample was reverse transcribed into cDNA with random hexamers following the manufacturer's instructions and purified with PCR cleanup columns (Qiagen 28115). SYBR green dye-based quantitative real-time PCR on a Quantstudio System 5 using mtrA-specific qPCR primers (5 μM), normalized to sigA, was performed. Expression was quantified by the ΔΔCt algorithm and data represent the mean ± s.d. for biological triplicates.

Antibiotic activity measurements

Isoniazid, vancomycin and cycloserine were dissolved in water, all other compounds were dissolved in DMSO. The CRISPRi-mediated *mtrA* knockdown strain (sgRNA1) and control sgRNA strain of *M. smegmatis* were pre-depleted in the presence of ATc (100 ng/ml) overnight and then spread on LB agar plates containing 100 ng/ml ATc. A filter disc with 10 μl of carbenicillin (100 mg/ml), isoniazid (0.5 mg/ml), vancomycin (6 mg/ml), cycloserine (100 mg/ml) or rifampicin (0.5 mg/ml) was placed in the center of plate and the diameter of inhibition of growth was measured after 4 days of growth. The CRISPRi-mediated *mtrA* knockdown strain (sgRNA5) and control NT sgRNA strain of *M. tuberculosis* were pre-depleted in the presence of ATc for 5 d before assay for IC_{50} analysis. Cultures were then diluted back to OD600 of 0.05 and 50 μl cell suspension was plated in technical triplicate in wells containing the test compound and fresh ATc. Compounds were dispensed into 384-well plate formal using an HP D300e digital dispenser. For compounds dissolved in water, 0.003% tyloxapol was added to help with dispensing. Plates were incubated standing at 37°C with 5% CO_2. OD600 was evaluated using a Tecan plate reader at 10 d post-plating and percent growth was calculated relative to the vehicle control for each compound. IC_{50} measurements were calculated using a nonlinear fit in GraphPad Prism. For all dose response curves, data represent the mean ± s.d. for technical triplicates. Data are representative of at least two independent experiments.

Time-kill assay

For each sample, *M. tuberculosis* CRISPRi-mediated *mtrA* knockdown strain (sgRNA5) were grown to mid-log phase in 7H9-rich media with Kanamycin (50 μg/ml) and then diluted back in the presence or absence of ATc to induce CRISPRi-mediated *mtrA* knockdown. Knockdown was allowed to proceed for 4 days. Antibiotic or vehicle control was added to the cultures and samples were collected at the designated time points, serially diluted and plated on 7H10 agar plates. Colonies were counted after 3 weeks. All time-kill assays were performed in biological triplicate and data are representative of two independent experiments.

QUANTIFICATION AND STATISTICAL ANALYSIS

Statistical analysis reported in this article were performed with GraphPad Prism, R or SciPy package in Python.[110] The details of p-values (e.g., test and sample parameters used) indicated in figures are described in figure legends. Statistically non-significant (NS) analysis results were considered with *p-value* > 0.05 and other qualifying *p-value*s were indicated accordingly * < 0.05, ** < 0.01, ***< 0.001, and ****<0.0001.

ADDITIONAL RESOURCES

All corem and GREs of the Mtb EGRIN 2.0 model are available on the Mtb web portal: http://networks.systemsbiology.net/mtb/.

Cell Reports, Volume *42*

Supplemental information

MtrA modulates *Mycobacterium tuberculosis* cell division in host microenvironments to mediate intrinsic resistance and drug tolerance

Eliza J.R. Peterson, Aaron N. Brooks, David J. Reiss, Amardeep Kaur, Julie Do, Min Pan, Wei-Ju Wu, Robert Morrison, Vivek Srinivas, Warren Carter, Mario L. Arrieta-Ortiz, Rene A. Ruiz, Apoorva Bhatt, and Nitin S. Baliga

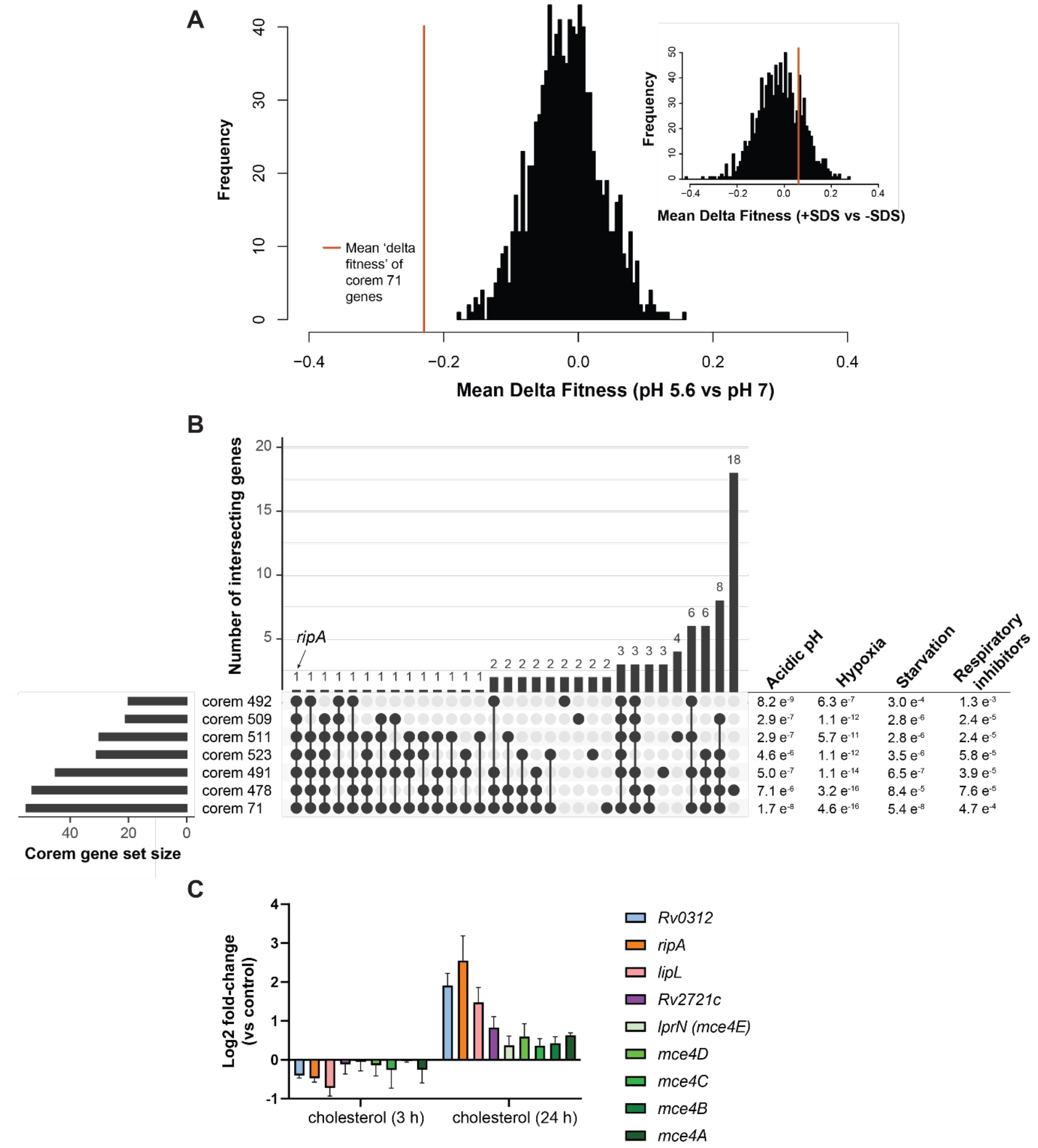

Figure S1. Environmental influences of corem 71. Related to Figure 2. (A) Histogram of mean delta fitness between acidic pH and neutral pH from 1000 permutations to generate shuffled gene sets. In each permutation, the produced shuffled gene set had the same size as corem 71. The orange line represents the observed value for corem 71. Inset displays the results of same analysis with mean delta fitness between the presence and absence of 0.05% SDS. (**B**) Upset plot of genes

in corems with similar environmental influences. Graph plots the number of genes in each corem and the intersection of genes within multiple corems. Each column corresponds to a single corem (single dot) or a set of corems (dots connected by lines below the X axis) containing the same genes. The number of genes in each set appears above the column, while the corems shared are indicated in the graphic below the column, with the corem numbers on the left. The enrichment (*P*-value) of environmental influences for each corem, calculated by hypergeometric test with BH correction is shown on the right. (**C**) Quantification (mean ± s.d., n = 3 biological replicates) of corem 532 genes' mRNA levels by microarray. H37Rv cells were grown in the presence of cholesterol for indicated time and compared to control. Data is from Yang Liu and Gary Schoolnik's unpublished carbon sources data.

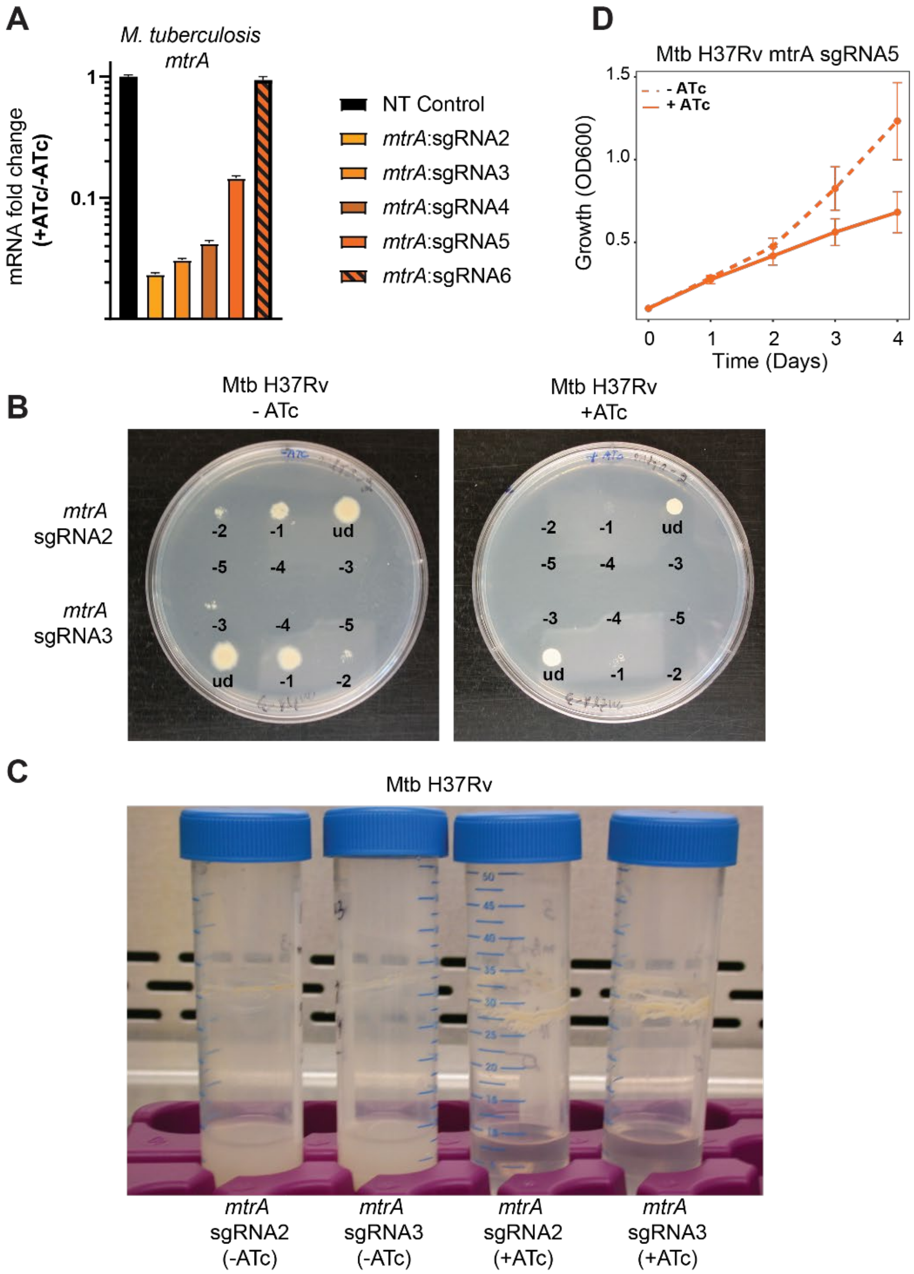

Figure S2. CRISPRi knockdown of *mtrA* with PAMs of various "strengths" in *M. tuberculosis*. Related to Figure 2. (**A**) Quantification (mean ± SD, *n* = 3 biological replicates) of *mtrA* mRNA levels by RT-qPCR. Strains were grown ± ATc for 4 days before collecting RNA. (**B**) Serial 10-fold dilutions of *M. tuberculosis* H37Rv CRISPRi strains with sgRNA2 and sgRNA3 targeting *mtrA* were spotted on 7H10 agar plates with (+ATc) or without ATc (-ATc control). (**C**)

Photographs of liquid cultures of the indicated strains. (**D**) Growth of *M. tuberculosis* H37Rv CRISPRi strain with sgRNA5 in liquid 7H9-rich media with (solid line) or without (dotted line) ATc. Growth was monitored daily by optical density at 650 nm. Points are the average of three biological replicates and error bars represent standard deviation.

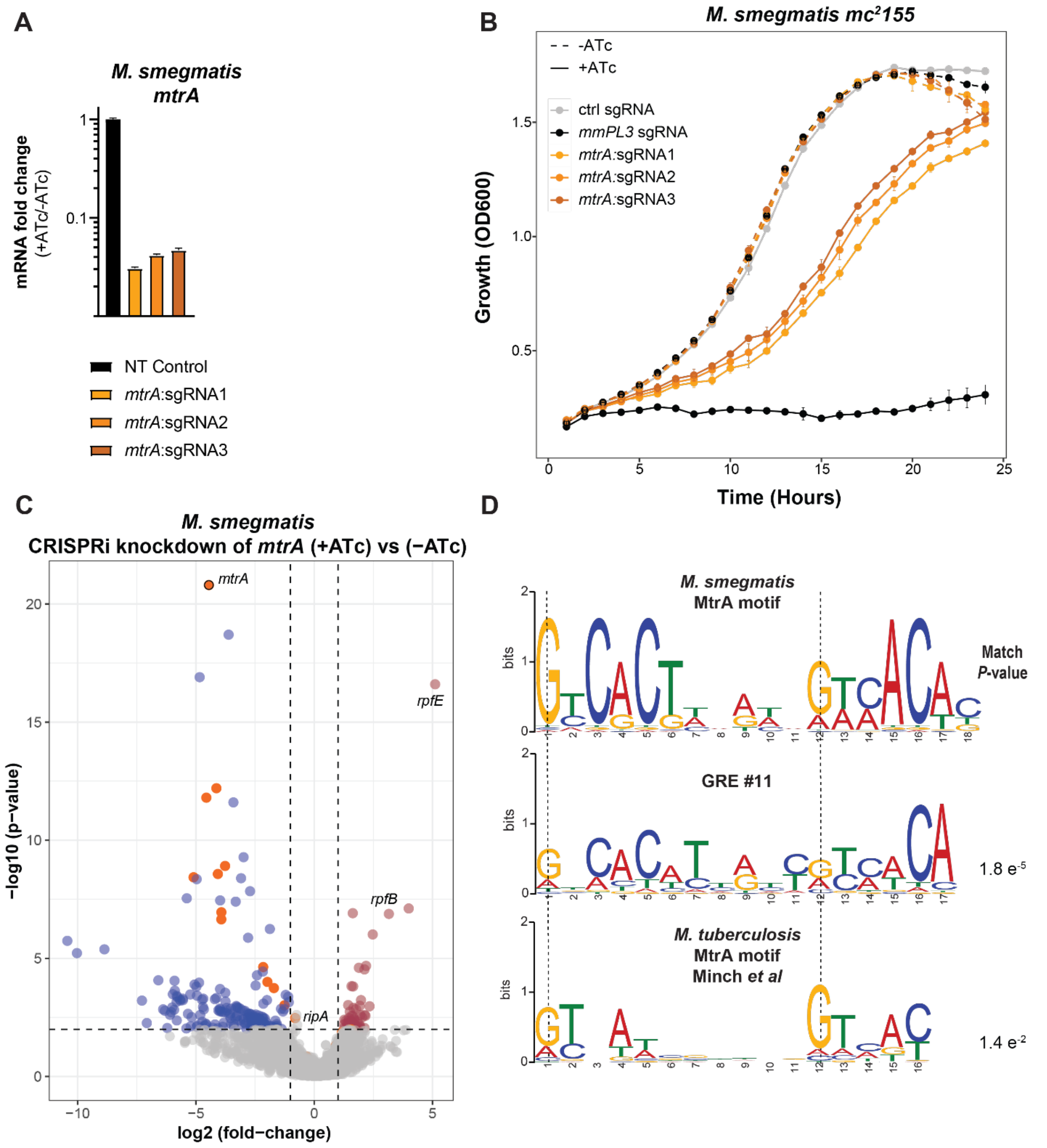

Figure S3. CRISPRi knockdown of *mtrA* in *M. smegmatis.* Related to Figure 2. (**A**) Quantification (mean ± SD, *n* = 3 biological replicates) of *mtrA* mRNA levels by RT-qPCR. Strains were grown ± ATc overnight before collecting RNA. (**B**) Growth of *M. smegmatis* mc^{2}155 CRISPRi strains with sgRNA1, sgRNA2, sgRNA3, control (ctrl) sgRNA, and sgRNA targeting

the essential gene *mmPL3* in liquid 7H9-rich media with (solid line) or without (dotted line) ATc. Growth was monitored hourly by optical density at 650 nm. Points are the average of three biological replicates and error bars represent standard deviation. (**C**) Volcano plot of differentially expressed genes for induced vs uninduced CRISPRi knockdown of *mtrA* in *M. smegmatis*. The significantly differentially expressed genes were selected by *p*-value < 0.01 and absolute log2 fold-change > 1. Dots represent different genes, with labels for particular genes of interest. Grey dots are genes without significant different expression, red dots are significantly up-regulated genes (*N* = 58 genes) and blue dots are significantly down-regulated genes (*N* = 185 genes). The orange dots are all genes of corem 71. (**D**) MEME analysis was performed on the promoter regions of candidate genes found to be significantly downregulated upon *mtrA* knockdown in Msm (*n* = 17 genes). The motif logo of MtrA from Msm compared to GRE #11 and MtrA motif deciphered through analysis of ChIP-seq mapped binding locations in Mtb [S1]. The *P*-values from alignment carried out with Tomtom [S2] are shown.

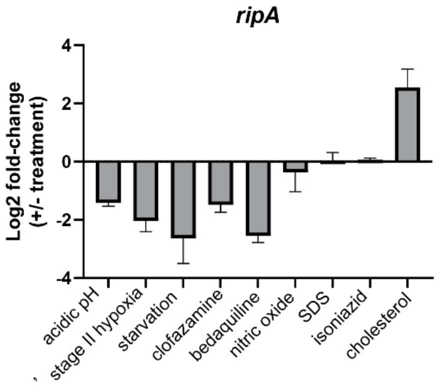

Condition	Description	Reference
acidic pH	pH 5.6 vs pH 7.0	This study
stage II hypoxia	0% dissolved oxygen, after 2 day slow/controlled oxygen depletion	PMID: 32348771
starvation	2 weeks in PBS-tyloxapol vs log growth	Baliga lab unpublished data
clofazamine	0.6 µg/mL clofazimine treated for 24 h vs untreated	GEO: GSE165673
bedaquiline	3.5 µg/mL bedaquiline treated for 24 h vs untreated	GEO: GSE165673
nitric oxide	1 mM DETA/nitric oxide for 24 h vs log growth	PMID: 28811595
SDS	0.05% SDS treated for 24 h vs untreated	Baliga lab unpublished data
Isoniazid	1.8 µg/mL isoniazid treated for 24 h vs untreated	GEO: GSE165673
cholesterol	1 mg/mL cholesterol for 24 vs control	Yang Liu and Gary Schoolnik's unpublished carbon sources data

Figure S4. Conditional expression of MtrA regulatory target, *ripA*. Related to Figure 2. The expression of *ripA* in conditions significantly enriched in corem 71 —acidic pH, hypoxia, starvation and treatment with respiratory inhibitors (*e.g.*, clofazimine and bedaquiline). Other conditions not enriched in corem 71 (*e.g.*, nitric oxide, SDS, and isoniazid treatment) are included for comparison. RipA is also found in corem 532 that is enriched in growth on cholesterol. Details of experimental conditions and data source are given in the table to the right.

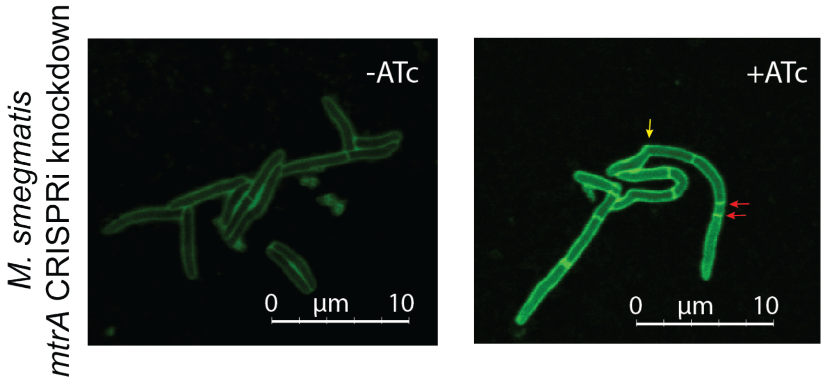

Figure S5. MtrA controls cell division in *M. smegmatis*. Related to Figure 4. Example micrographs of uninduced (-ATc) and induced (+ATc) CRISPRi knockdown of *mtrA* with sgRNA1 in Msm. After knockdown, cells were labeled with HCC-amino-D-alanine (HADA) for 3.5 h. Red arrows indicate multiple septa and yellow arrow indicates the curved shape phenotype. Data are representative of at least two independent experiments.

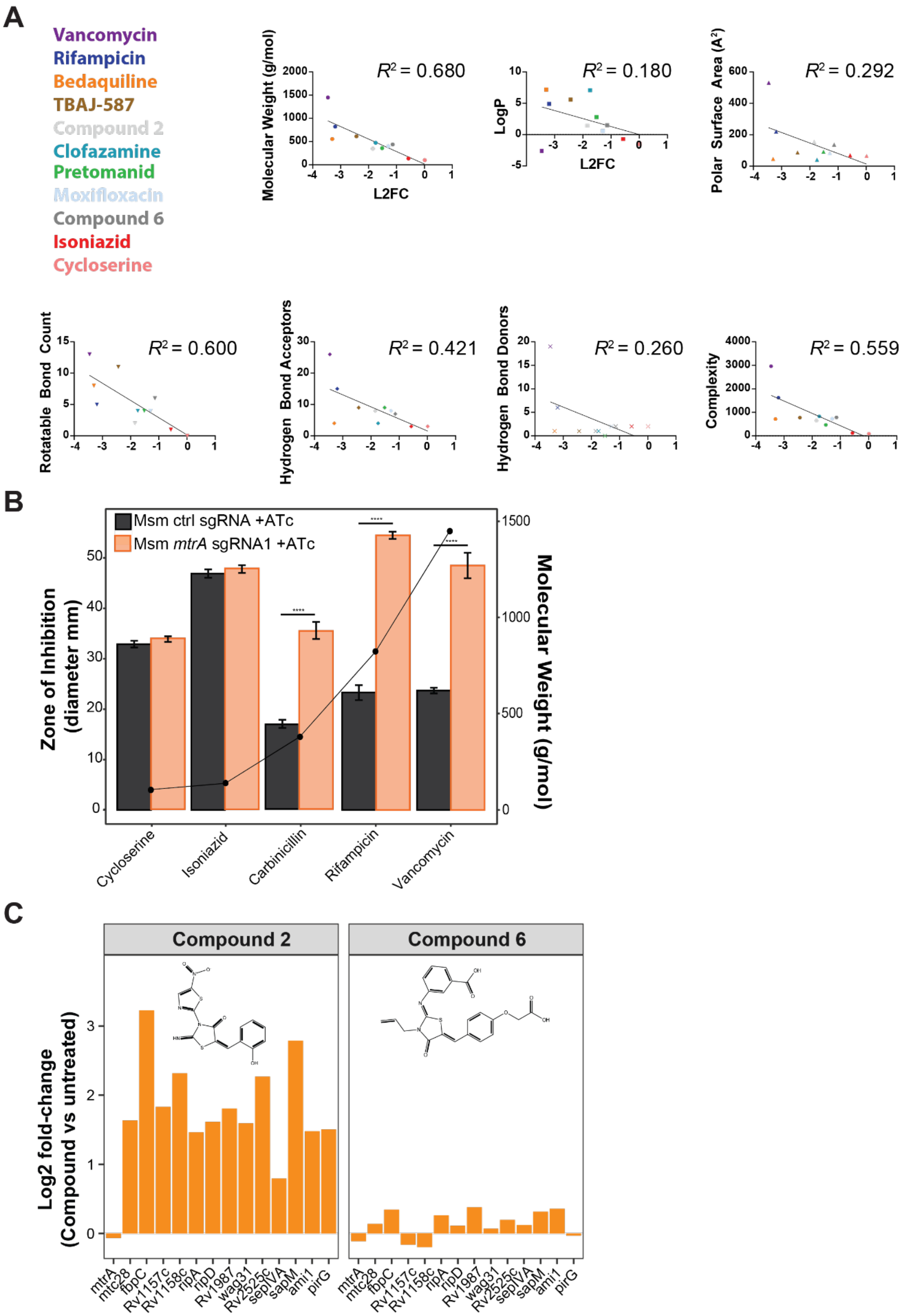
A
Vancomycin
Rifampicin
Bedaquiline
TBAJ-587
Compound 2
Clofazamine
Pretomanid
Moxifloxacin
Compound 6
Isoniazid
Cycloserine
Molecular Weight (g/mol)
R^2 = 0.680
L2FC
LogP
R^2 = 0.180
L2FC
Polar Surface Area (Å²)
R^2 = 0.292
Rotatable Bond Count
R^2 = 0.600
Hydrogen Bond Acceptors
R^2 = 0.421
Hydrogen Bond Donors
R^2 = 0.260
Complexity
R^2 = 0.559
B
Msm ctrl sgRNA +ATc
Msm *mtrA* sgRNA1 +ATc
Zone of Inhibition (diameter mm)
Molecular Weight (g/mol)
****
Cycloserine
Isoniazid
Carbinicillin
Rifampicin
Vancomycin
C
Compound 2
Compound 6
Log2 fold-change (Compound vs untreated)
mtrA
mtc28
fbpC
Rv1157c
Rv1158c
ripA
ripD
Rv1987
wag31
Rv2525c
sepIVA
sapM
ami1
pirG

Figure S6. Antibiotic activity with *mtrA* knockdown. Related to Figure 5. (**A**) For each drug, the Log2 fold-change (L2FC) in IC_{50} for *mtrA* knockdown in Mtb compared to control (NT sgRNA) was compared to various physiochemical properties. The IC_{50} measurements were calculated using a nonlinear fit and the R^2 values were determined using linear regression in GraphPad Prism. (**B**) The induced CRISPRi *mtrA* knockdown with sgRNA1 and control (ctrl) sgRNA strains of Msm were spread on LB plates containing 100 ng/ml ATc inducer in triplicate. A filter disc with 10 µl antibiotic was place in the center of plate and the diameter of inhibition of growth was measured after 4 days of growth. Antibiotic concentration on disc: cycloserine (100 mg/ml); isoniazid (0.5 mg/ml); carbinicillin (100 mg/ml); rifampicin (0.5 mg/ml); vancomycin (6 mg/ml). Significance was determined by Student's T-test. ****: p-value < 0.0001. Results are mean from technical triplicates. All data are representative of at least two independent experiments. (**C**) Mtb H37Rv was treated with 34 µg/mL of Compound 2 or 80 µg/mL of Compound 6 for 72 h. Cell pellets were collected for RNA extraction and mRNA was quantitated by RNA-seq.

Table S1. **sgRNAs used in this study**.

Organism	Gene	sgRNA name	Forward	Reverse	PAM (5'-3')
M. smegmatis	*mtrA*	sgRNA1	GGGAGGTGTCGAAACCCTCACCAC	AAACGTGGTGAGGGTTTCGACACC	AGAAC
M. smegmatis	*mtrA*	sgRNA2	GGGAATCGATGCCGTTCATCCCAG	AAACCTGGGATGAACGGCATCGAT	AGCAT
M. smegmatis	*mtrA*	sgRNA3	GGGAGACGGTGTCGGTCTTGGCGG	AAACCCGCCAAGACCGACACCGTC	AGCAT
M. smegmatis	NT	control_sgRNA	GGGAGGAGACGATTAATGCGTCTCG	AAACCGAGACGCATTAATCGTCTCC	AGAAA
M. smegmatis	*mmpL3*	mmpL3	GGGAGCGACAGACTGGCTGCCCTCGTC	AAACGACGAGGGCAGCCAGTCTGTCGC	AGAAA
M. tuberculosis	*mtrA*	sgRNA2	GGGAATCCACGGTGTCGGTCTTTGCGG	AAACCCGCAAAGACCGACACCGTGGAT	AGCAT
M. tuberculosis	*mtrA*	sgRNA3	GGGAGATTTCTACGTCGGCGATGG	AAACCCATCGCCGACGTAGAAATC	AGCAT
M. tuberculosis	*mtrA*	sgRNA4	GGGAGTCTTTGCGGTGAGCATCACGA	AAACTCGTGATGCTCACCGCAAAGAC	GGAAC
M. tuberculosis	*mtrA*	sgRNA5	GGGAGCCGGTAACCCCATACCTGTT	AAACAACAGGTATGGGGTTACCGGC	AGCAG
M. tuberculosis	*mtrA*	sgRNA6	GGGAGTCAGCACCACAGTCGGGTTC	AAACGAACCCGACTGTGGTGCTGAC	GGGAT
M. tuberculosis	NT	control_sgRNA	GGGAGGAGACGATTAATGCGTCTCG	AAACCGAGACGCATTAATCGTCTCC	AGAAA

Table S2. Genes with predicted regulation by MtrA.

Gene	Name	Description	Gene with reduced fitness at acidic pH	Gene with GRE #11 in promoter	Mtb *mtrA* knockdown +Atc vs –ATc Log2 FC (p <0.01)	Mtb *mtrA* knockdown Li *et al* Log2 FC (p <0.01)	Msm homolog	Msm *mtrA* knockdown +Atc vs –ATc Log2 FC (p <0.05)
Rv3246c	*mtrA*	essential response regulator			-3.76	-1.21	*MSMEG_1874*	-4.56
Rv0040c	*mtc28*	secreted protein	X	X	-2.99	-3.58	***MSMEG_6919***	**-4.13**
Rv0129c	*ag85C/fbpC*	cell wall trehalose dimycolate biosynthesis	X	X	-1.59	-2.6	***MSMEG_3580***	**-3.77**
Rv0179c	*lprO*	lipoprotein		X	-1.08	-1.5	***MSMEG_0210***	**-1.7**
Rv0312		proline and threonine rich		X	-2.68	-4.66	*MSMEG_0638*	ns
Rv1157c		conserved protein		X	-1.24	-0.85	***MSMEG_5153***	**-4.45**
Rv1158c		conserved protein	X	X	-1.9	-2.71	***MSMEG_5151***	**-5.1**
Rv1435c		proline, valine, glycine rich secreted		X	-2.85	-2.24	*MSMEG_0673*	-3.93
Rv1477	*ripA*	peptidoglycan endopeptidase	X	X	-2.4	-1.81	***MSMEG_3145***	**-0.80**
Rv1566c	*ripD*	peptidoglycan endopeptidase		X	-2.54	-2.17	*MSMEG_3477*	-3.1
Rv1690	*lprJ*	lipoprotein		X	-1.72	-2.55	***MSMEG_4689***	**-4.07**
Rv1987		probable chitinase		X	-3.3	-2.21		
Rv2145c	*wag31*	essential cell elongation protein DivIVA		X	-1.2	-0.66	*MSMEG_4217*	ns
Rv2525c		peptidoglycan glycoside hydrolase		X	-1.07	-0.69	*MSMEG_6815*	ns
Rv2894c	*xerC*	tyrosine recombinase		X	-2.26	-1.68	***MSMEG_2515***	**-1.26**
Rv2927c	*sepIVA*	essential cell septation protein		X	-1.2	ns	***MSMEG_2416***	**1.11**
Rv3310	*sapM*	acid phosphatase	X	X	ns	-1.24		
Rv3717	*ami1*	peptidoglycan amidase	X	X	-1.75	-1.46	***MSMEG_6281***	**-3.92**
Rv3810	*pirG/erp*	exported repetitive protein	X	X	-3.0	-1.67	***MSMEG_6405***	**-1.98**
Rv1075c		conserved exported protein			-1.82	-1.53	*MSMEG_5272*	ns
Rv1076	*lipU*	lipase			-4.91	-3.11	*MSMEG_5271*	ns
Rv1469	*ctpD*	cation transporter P-type ATPase		*	-2.00	-1.51	*MSMEG_5403*	-1.55
Rv1478	*ripB*	peptidoglycan endopeptidase	X		-3.13	-2.37	*MSMEG_3146*	-1.20
Rv1754c		conserved protein			-2.92	-1.78	*MSMEG_2107*	-3.62
Rv3229c	*desA3*	stearoyl-CoA 9-desaturase			-1.03	-1.34	*MSMEG_1886*	-0.93

Msm homologs in bold have significant MtrA binding site in their promoter. Genes shaded in grey are not part of corem 71.

Supplemental Information References

S1 Minch, K.J., Rustad, T.R., Peterson, E.J., Winkler, J., Reiss, D.J., Ma, S., Hickey, M., Brabant, W., Morrison, B., Turkarslan, S., et al. (2015). The DNA-binding network of Mycobacterium tuberculosis. Nat Commun *6*, 5829. 10.1038/ncomms6829.

S2 Gupta, S., Stamatoyannopoulos, J.A., Bailey, T.L., and Noble, W.S. (2007). Quantifying similarity between motifs. Genome Biol *8*, R24. 10.1186/gb-2007-8-2-r24.
